# Perennial Rye: Genetics of Perenniality and Limited Fertility

**DOI:** 10.3390/plants10061210

**Published:** 2021-06-14

**Authors:** Paul Gruner, Thomas Miedaner

**Affiliations:** State Plant Breeding Institute, University of Hohenheim, 70593 Stuttgart, Germany; Paul.Gruner@uni-hohenheim.de

**Keywords:** *Secale cereale*, *Secale montanum*, *Secale strictum*, QTL mapping, molecular marker, self-incompatibility, fertility, seed set

## Abstract

Perenniality, the ability of plants to regrow after seed set, could be introgressed into cultivated rye by crossing with the wild relative and perennial *Secale strictum*. However, studies in the past showed that *Secale cereale × Secale strictum*-derived cultivars were also characterized by reduced fertility what was related to so called chromosomal multivalents, bulks of chromosomes that paired together in metaphase I of pollen mother cells instead of only two chromosomes (bivalents). Those multivalents could be caused by ancient translocations that occurred between both species. Genetic studies on perennial rye are quite old and especially the advent of molecular markers and genome sequencing paved the way for new insights and more comprehensive studies. After a brief review of the past research, we used a basic QTL mapping approach to analyze the genetic status of perennial rye. We could show that for the trait perennation 0.74 of the genetic variance in our population was explained by additively inherited QTLs on chromosome 2R, 3R, 4R, 5R and 7R. Fertility on the other hand was with 0.64 of explained genetic variance mainly attributed to a locus on chromosome 5R, what was most probably the self-incompatibility locus *S5*. Additionally, we could trace the *Z* locus on chromosome 2R by high segregation distortion of markers. Indications for chromosomal co-segregation, like multivalents, could not be found. This study opens new possibilities to use perennial rye as genetic resource and for alternative breeding methods, as well as a valuable resource for comparative studies of perennation across different species.

## 1. Introduction

In 2019, rye was grown on about 4.2 million hectares worldwide resulting in an overall production of about 12.8 million tons [1]. This data, however, must be mainly based on annual rye (*Secale cereale* L.) because only a limited number of perennial rye (*Secale cereale × Secale strictum*) cultivars were available and to our knowledge the last breeding efforts were done more than a decade ago. According to the original habitat of *Secale strictum* Presl. (syn. *Secale montanum* Guss.) in dry, stony or sandy mountain areas or as weed in or along cultivated fields [2], the breeding goal for perennial rye was to develop cultivars for poor and sandy soils that could be used as forage [3,4,5] or as soil cover and (especially winter) pasture for solely grazed areas [6] or combined as fodder and grain crop in low-input farming [7,8]. Beside high drought (and cold) tolerance more traits of *S. strictum* were interesting for breeding: large root systems, high tillering capacity, weed suppression, pest resistances, tolerance to (heavy) metals like nickel, zinc, aluminum and manganese and high protein content of kernels [2,9,10,11].

*Secale cereale × Secale strictum* crosses aiming for establishing perennial rye cultivars were made by several groups worldwide. Reimann-Phillipp in Germany released two tetraploid varieties ‘Permontra’ (winter type) and ‘Sopertra’ (spring type) and produced diploid breeding material that did not reach official variety registration [8], but was used in this study. Kruppa and Kotvics in Hungary released the varieties ‘Kriszta’ and ‘Perenne’ [3,5] and Myers in Australia produced the (non-registered) cultivar ‘Black Mountain’ [6]. The Letbridge Research and Developmental Centre in Canada developed the cultivar ‘ACE-1’ based on selections derived from Reimann-Philipp [4].

**Figure 1:**
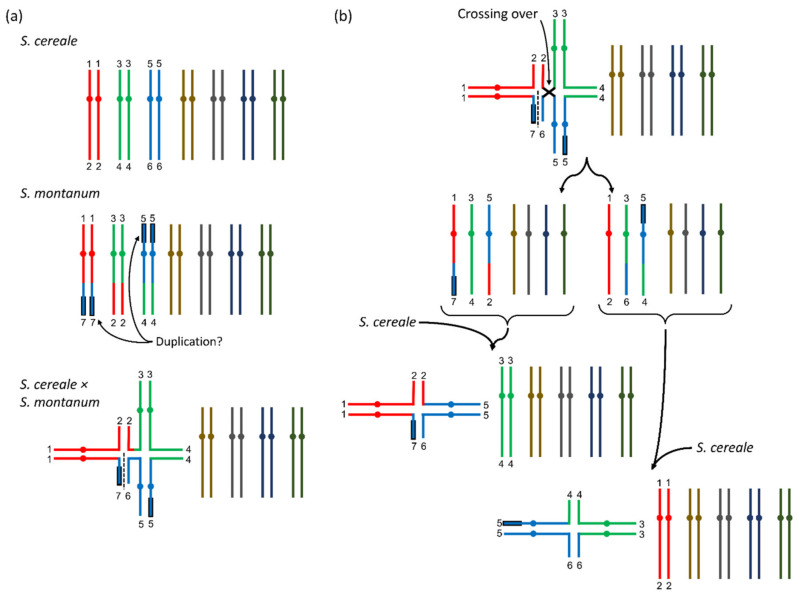
Visualization of multivalents in *S. cereale* × *S. strictum* progenies according to results from Stutz [17]. (**a**) In *S. cereale × S. strictum* a multivalent of six chromatids could be detected in metaphase I of pollen mother cells. This was caused by translocations between three chromosomes of the two parental species (in color). Following [17], the numbers indicate the chromosomal assignment, the black framing of the chromosomes indicates a potential duplication and the dashed line the separation point for chromosomal line configurations that were also observed. (**b**) In an F_2_-generation, in rare cases also multivalents with four chromatids could be observed. This could be explained by a crossing over within the ring conformation (top). When the resulting gametes (two possible configurations) were then combined with *S. cereale* gametes, two differently composed four-chromosome multivalents could be formed and another two different compositions would be possible when they would be combined with the S. strictum gametes. Here, only the combination with *S. cereale* gametes is shown and more details can be found in [17].

Compared to the annual *S. cereale*, all breeding efforts were confronted with fragile rachis (brittle ear), low grain yields, loose stands especially in the years after first harvest, low fertility, long periods of flowering and ripening and high ergot infections arising as consequence of the latter two in combination with wet weather conditions [4,5,6,7,8,10]. Crossing barriers between *S. cereale* and *S. strictum* were the main reason for the low fertility and cytological studies of pollen mother cells (PMCs) revealed abnormalities. In metaphase I (or anaphase) of the PMCs, where the sister chromatids usually pair together (= seven bivalents), often a multivalent of six chromatids (= three chromosomes in ring or line formation) plus four bivalents (= four chromosomes) was found and this indicated (two) translocations on the three multivalent-forming chromosomes (Figure 1a) [12,13,14,15,16].

With low frequency, even more chromosomal constitutions could be identified in the progenies [12,13,14,16], however, some of the results had to be questioned [17] because *S. strictum* accessions that were used showed multivalents also for plants of wild *S. strictum*, which was in disagreement with the other authors listed before. The phenomenon of multivalent formations and explanations for the different constitutions were best described in Stutz [18] and Dierks and Reimann-Philipp [19]. The important link between low fertility and chromosomal translocations was the assumption that DNA of all seven chromosomes was required for a viable gamete. The arbitrary segregation of the six multivalent chromosomes would only result in fertile gametes when all three chromosomes of a single parental species would segregate together. If the arbitrary segregation of the chromosomes of the multivalent was considered, Dierks and Reimann-Philipp [19] calculated that functional chromosomal constitutions could be expected in 25 percent of the cases only. Additional fertility problems of *S. strictum* and potential fertile abnormal chromosomal constitutions may had influenced this theoretical ratio in a way that fertility assessment of microgametes (pollen) and macrogametes (kernels/inflorescences) often exceeded the theoretical expectation calculated from chromosomal segregation ratios [12,19].

Aside of cytogenetically caused non-fertility in the *S. cereale × S. strictum* progenies, we added another fertility-related factor in our experiment by crossing a self-incompatible *S. cereale × S. strictum* genotype with a self-fertile inbred line. The cultivated rye *S. cereale* is generally a self-incompatible cross-pollinating species, but self-fertile genotypes (resulting in inbred lines) were developed by recurrent selection of partially self-fertile plants detected in extremely large populations (N > 50,000) [20]. The self-incompatibility in rye has been referred to gametophytic mechanisms and interaction of two loci named *S* and *Z* [21] and two loci on chromosome 1R and 2R have been referred to those genes [22,23,24]. The genes for self-fertility (or pseudocompatibility) have been referred to the same loci [25,26] and self-fertility can be interpreted as special allele *Sf* of the self-incompatibility locus [27,28]. Aside of the *S* and *Z* loci (= *S1* and *S2* loci), a further self-fertility locus *S5* was located on chromosome 5R [28,29]. Even more dominant self-fertility genes were found on chromosomes 1R, 4R, 5R and 6R [29].

Based on microscopic studies, the chromosomes involved in multivalent formation were referred to 2R, 6R and 7R [8] and due to the reasons listed before, the identification of any gene thereon would be challenging. For the perennial habit (perenniality), Dierks and Reimann-Philipp [19] concluded that a major (dominant) gene *P* was located on one of the respective chromosomes. The fertility problems of all the breeding attempts with *S. cereale × S. strictum* progenies did support this hypothesis and the only disagreement with this theory coming from Stutz [18], could be regarded to the misinterpretation of the perennial phenotype. Other traits like the fragile rachis (“brittle ears”) typical for *S. strictum*, or spring/winter type (vernalization requirement for flower induction) when crossed with spring-type *S. cereale* were inherited independently from the multivalent according to Dierks and Reimann-Philipp [19], however, also close correlation of perenniality and fragile rachis had been observed in experiments where only 1% of the F_2_-plants showed perenniality without fragile rachis [20].

Nevertheless, recombination was also observed between the homologous chromosomal segments of the multivalent. Thus, if the perenniality gene *P* would be located at a (distal) chromosomal position where still recombination occurred, it must be possible to identify perennial *S. cereale* recombinants. If there would be no crossing over between a *P*-carrying *S. strictum* chromosome and a *S. cereale* chromosome resulting in viable gametes, the mapping of the perenniality gene would be impossible with classical crossing experiments and consequently, the breeding of fertile perennial rye varieties would require homozygosity for all three translocated *S. strictum* chromosomes [18,19]. Even further, any introgression of *S. cereale* would disturb this configuration and lead again to a high percentage of non-viable gametes and hence reduced fertility. However, Stutz [18] found another multivalent constitution by analyzing a F_2_ generation. There, single genotypes showed pollen mother cells with only four chromatids (=2 chromosomes) and five remaining bivalents in metaphase I. This could be explained by a crossing over across the six-chromatid ring multivalent in the F_1_ plants (Figure 1b). These configurations would be especially valuable for genetic studies and breeding, because two possible configurations of four-chromosome multivalents with different chromosomes involved could theoretically occur (Figure 1b) and thus it would allow to identify the respective chromosomes and with further crossing and recombination steps also the gene loci.

To our knowledge, the last genetic studies of perennial rye were at the methodological level of chromosome microscopy, but perenniality (and fertility) was also studied in other cereals (rice, sorghum, maize, wheat) and their perennial wild relatives by means of molecular markers [30,31,32,33,34,35,36] and sequence based genomics and transcriptomics [37,38,39,40]. All those studies showed that the genetics of perenniality was highly complex. Several QTLs and even more gene candidates, of which often several could be located in one single QTL were found. Interestingly, almost all loci that were identified independently in the separate species could be connected through syntenic gene motifs (DNA and protein sequences) across the species, showing that this trait was highly conserved between the species.

For this study, a F_2_ population originating from crossing an annual and self-fertile inbred line with a perennial and self-incompatible plant from an improved perennial population that was originally derived from breeding material of Reimann-Philipp [8] was phenotypically assessed for perenniality and fertility and genomically with an Infinium iSelect 10K SNP-chip. At first, we present the phenotypes for both traits separately and the relation between them, then results from studying the pure marker data in regard to multivalents and other abnormalities and finally the QTL-mapping results for both traits.

## 2. Results

### 2.1. Phenotype

**Figure 2:**
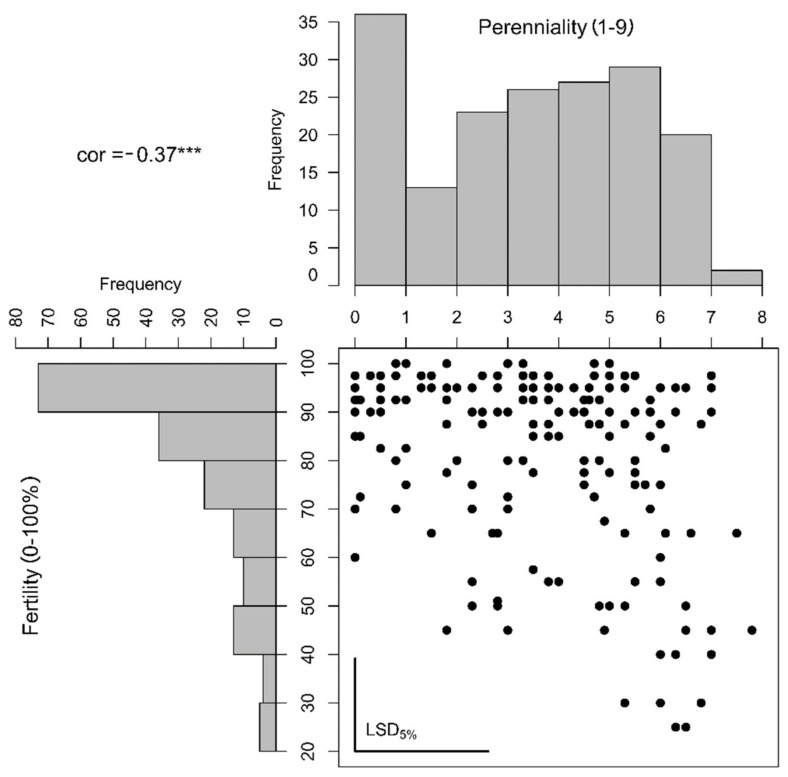
Histograms and correlation plot based on the estimated genotypic means (best linear unbiased estimators, BLUES) of perenniality (scored from 1 to 9) and non-fertility (scored from 0 to 100%). The calculated correlation (cor) was estimated to be −0.37 and highly significant with α ≤ 1% (***). The least-significant differences (LSD) on α ≤ 5% significance level were plotted as bars in the bottom-left corner of the correlation plot.

For both traits, perenniality and fertility, a high amount of genetic variance was observed (Table 1). Perenniality (1–9) was assessed at two sites, but the maximum value of 9 was only recorded once for a single plant and the maximum calculated BLUE was 8 (Figure 2). Most of the phenotypic variance was explained by the genotype, but also the genotype–location interaction was high (Table 1).

The lowest observation for fertility was 10%, but only a few plants showed this low fertility and all estimated BLUES were higher than 20% (Figure 2). Most of the phenotypic variance was explained by the genotype but no genotype–location interaction could be calculated. For both traits, the entry-mean heritability was high (0.81 and 0.87) but the phenotypic distributions were non-normal (Figure 2). Correlation between both traits was only moderate (0.37). This and the observation of highly perennating genotypes combined with high fertility (Figure 2) indicated an incomplete linkage of both traits. The opposite off-correlation extreme, no perenniality and low fertility, could not be found indicating that still the annual genotypes were the most fertile.

### 2.2. Marker Studies

**Figure 3:**
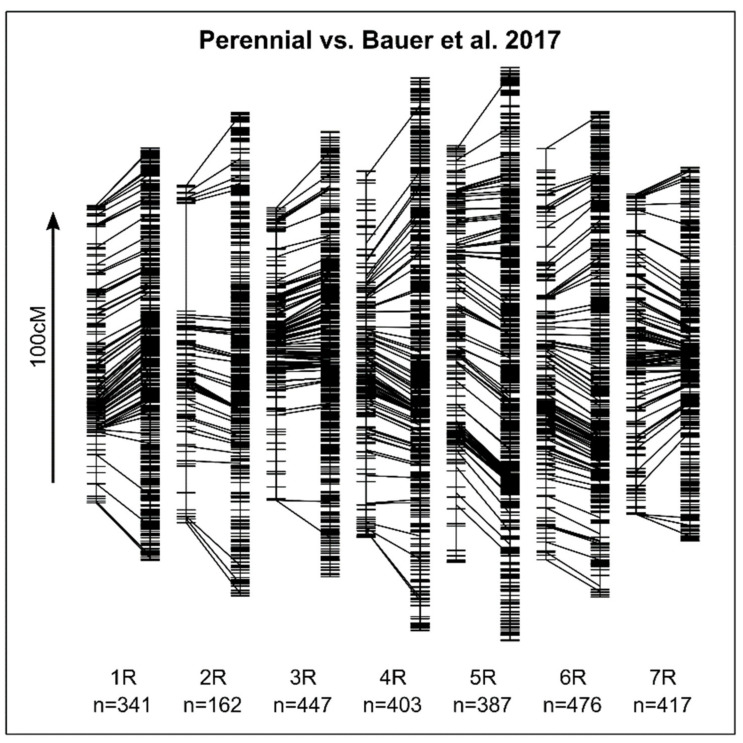
Chromosome-wise comparison of a linkage map constructed from the marker data analyzed here (Perennial, left) with a previously published linkage map [41] (right). Overlapping markers of the respective linkage groups (1R–7R) are connected by lines. For comparisons of the absolute sizes in cM, an arrow indicating the chromosomal order with a length of 100 cM is displayed in the left of the plot. The number of markers in each linkage group of the constructed linkage map (Perennial) is reported (n).

The study of pure marker data had two purposes. Firstly, this was the basis for QTL mapping and secondly, we could use it to find indications for chromosomal abnormalities like multivalents that would had directly influenced fertility. We could successfully apply the Infinium iSelect SNP chip that was initially developed for *S. cereale*, for a *S. cereale* × *S. strictum* population. After filtering, 2641 markers remained from the 10K SNP assay and entered into the analysis. Caused by the few possible recombination events generally observable in F_2_-generations, 2314 markers were intercorrelated with one (=redundant), many in several combinations, resulting in 789 unique markers. For those, the “A” marker allele had a frequency of 25.5% on average, the “B” allele of 22.8%, the heterozygous of 51.5% and the missing values 0.1%. A linkage map could be constructed and except for some chromosomal regions, it covered the full genome when compared with overlapping markers (n = 812) from a previously published linkage map [41] (Figure 3). Some chromosomal regions (on 2R, 3R, 5R and 7R) were less covered with molecular markers compared to the previously published map (Figure 3). However, the map from Bauer et al. [41] had many more markers (N = 87,820) and no (large) differences in the marker order of both maps were observed so that our map was still considered sufficient for the linkage mapping.

**Figure 4:**
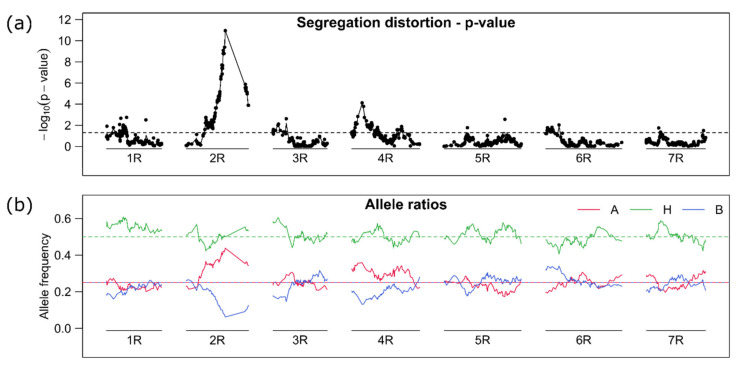
(**a**) Test of markers allele frequencies for distortion from the expected 1:2:1 (A, H, B) ratio of markers on the seven linkage groups (1R–7R). The –log_10_ of a Chi-square test is displayed. The horizontal dashed line gives a (unadjusted) global 5% threshold level. (**b**) The allele frequency for the respective marker alleles (A, H, B) are displayed (red, green, blue) along the chromosomes. The horizontal lines give the expected frequencies 0.25 for the homozygous state (A, B) and 0.5 for the heterozygous state (H). The lines were drawn by connecting single marker-based estimates.

The largest gap on the top arm of chromosome 2R was also characterized by high segregation distortion, where the “B” marker alleles were reduced to zero resulting in a 1:1 ratio for the “A” and “H” allele. The full reduction of the “B” marker allele at zero could not be visualized in Figure 4, because we filtered the marker data set in a first step (before linkage map construction). If we used a linkage map based on other material like the one from Bauer et al. [41], the gap would be flanked by the markers isotig16940 at 114.7cM and C3277_855 at 124.3 cM. We referred this locus to the self-incompatibility locus *Z* [23,24] what is probably equal to the self-fertility locus *S2* [25,26]. The missing of the “B” allele and segregation ratio of 1:1 for the “A” and “H” allele agreed well with a self-fertility model with pollen compatibility [28] and previously reported segregation ratios [25]. Segregation distortion to a lower extend could also be observed at the top arms of chromosome 4R and 6R, but it was considered irrelevant for following results. The successful construction of a linkage map was also an indicator that the linkage of markers was not influenced by chromosomal segregation abnormalities like multivalents. We further correlated all markers with each other and did not find any high correlation between markers of certain chromosomes (Figure S1) like it would be expected if only a certain parental chromosome (chromatid) combination would result in fertile gametes as proposed for example by Stutz [18] (Figure 1a).

### 2.3. Mapping

To detect markers associated with perenniality or fertility, we tested different procedures (scans) without marker cofactors and with differently selected cofactors and results were presented in Table 2 and Figures S2–S7, marker sequences can be found in Table S1. The cofactors and cofactor selection methods only marginally influenced the mapping results. Difference in detected loci between the methods could only be observed for the markers that explained the least variance in single marker fits (QTL-F1a, QTL-F1b, QTL-P2, QTL-P2).

**Figure 5:**
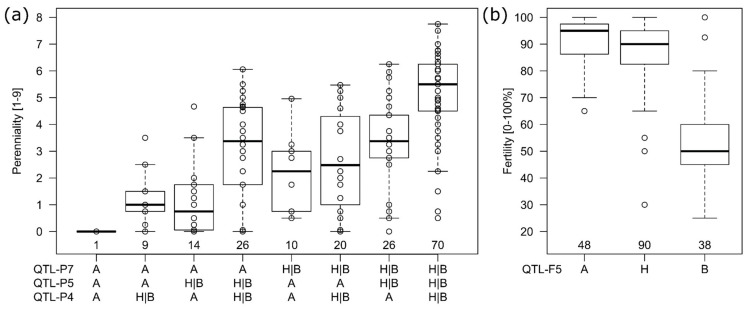
Genotypic means for perenniality (**a**) and fertility (**b**) clustered by marker alleles A, H and B (*x*-axis) of the most significant markers in the respective QTLs. For perenniality (**a**) the H and B allele was combined in one group (H|B) and all genotype means were additionally plotted as dots in the boxplot display (box at first and third quartile with median in center). The number of genotypes with the respective marker allele combinations is written above the *x*-axis within the plot.

For perenniality, three QTL were consistently found by all methods (Table 2). They were located on chromosome 4R (QTL-P4), chromosome 5R (QTL-P5) and chromosome 7R (QTL-P7). Those QTLs also explained most of the genetic variance, each 0.16, 0.23 and 0.24, respectively. The effects for the first two were mainly codominant with effect sizes of 1.15 and 1.34, whereas the latter was dominant (d effect = cd effect) or even over-dominant (d effect > cd effect), i.e., having a codominant effect of 0.64 and a dominant effect 1.61. All QTL (QTL-P2 to QTL-P7) were highly additive as the explained genetic variance of a model with markers from all QTL together was 0.74 and exactly the same as when explained variances of the single marker fits would be summed up. When combinations of the three QTLs explaining most of the variance (QTL-P4, QTL-P5, QTL-P7) were compared (Figure 5a), the genotypes having all three wild type alleles (H or B) had the highest perenniality followed by genotypes with two wild type alleles of which the combinations of QTL-P4 with QTL-P5 and QTL-P5 with QTL-P7 were on average higher than the combination of QTL-P4 with QTL-P7 (Figure 5a). However, for all combinations also genotypes with only little or even no perennation could be found (Figure S8).

The trait fertility was mainly explained by a major QTL (QTL-F5) on chromosome 5R explaining 0.64 of the genetic variance (Table 2, Figure 5b). The codominant effect was −18.4 and the dominant effect with 14.9 almost as high as the codominant. The negative effect size indicated that the A allele (inbred line) was leading to higher fertility (Figure 5b). More QTLs (QTL-F1a, QTL-F1b, QTL-F4) on chromosome 1R and 4R could be found. The additional QTLs explained only 0.06 to 0.13 of the genetic variance, had smaller (negative) effect sizes and the (positive) dominant effects of QTL-F1a and QTL-F1b again indicated the fertility to be dominantly inherited by the parental A allele for those two loci.

Additionally to this basic QTL mapping approach, we studied epistatic effects in terms of marker–marker interactions (Figures S9–S14) and to detect those, all possible combinations with cd-cd, d-d and cd-d marker interactions were tested in a model with the same markers as single (cd and d) main effects simultaneously. As this resulted in many more tests, we adjusted the global significance threshold. With this stricter threshold no significant marker–marker interactions could be found, except of some single neighboring markers on chromosome 4R with cd-d interaction being significant. Nevertheless, for both traits several chromosomal regions showed high *p*-values for marker–marker interactions, but only a few were associated with the main QTLs we found. We concluded that with the given study set-up (population size and phenotypic error) we could not infer any useful conclusion regarding epistasis. Anyway, a detailed visualization can be found in Figures S9–S14. We also estimated explained covariance of both traits for the respective QTLs, but the explained covariance estimated for each QTL (Table 2) was generally low and the largest influence on the explained genetic covariance for a single marker were QTL-F4, QTL-F5, QTL-P4 and QTL-P5 with values between 0.25 and 0.39 each.

## 3. Discussion

Our study revealed new insights into perenniality and fertility of *S. cereale* × *S. strictum* progenies and we could show that perenniality was a complex trait with several QTLs involved compared to fertility (or non-fertility) which was mainly caused by a self-fertility allele at a self-incompatibility locus coming from the perennial parent. We could not find any indications for abnormalities in chromosomal segregation (multivalents) and their relation to low fertility, but as this was intensively discussed in previous studies we will discuss it in more detail here. It will be followed by a discussion of the results from mapping fertility and perenniality. Due to the scarce genomic-related literature on perenniality in rye, we addressed the usefulness of results from other species (species sytheny) and finally ended in future breeding perspectives.

### 3.1. Multivalents

We could not find an ultimate explanation why we could not prove the presences of multivalents with molecular markers. It was most reasonable, that the perennial genotype that we had used as parent did not result in multivalents (anymore), because it belonged to an improved perennial population from Reimann-Phillipp, who continued (also privately) with perennial rye breeding after releasing his research works on this topic and his major selection criterium was high fertility. Stutz [18] showed a possible way out of multivalents (Figure 1b) where crossing overs between the chromosomes could reduce the initial six-chromatid multivalent into a four-chromatid multivalent and it may be possible that following recombination events would have even led to chromosomal constitutions without any multivalent. Additionally, recombination was observed between the chromosomes of the multivalent [18,19] what additionally could had caused translocated chromosomal segments to become smaller and reduced the lethality for gametes with certain combinations of translocated chromosomes. Even further, not only the genetic resource itself but also the perennial F_1_ plant was chosen based on high fertility from crosses that had been made with several perennial plants to develop the population under study. Even more could the (indirect) selection against the “B” allele at the self-incompatibility loci *Z* have influenced the constitution of the respective population as it would be located on one of the multivalent forming chromosomes [8]. To further clarify this issue, again the chromosomes in metaphase I of the pollen mother cells must be (microscopically) studied. Unfortunately, the material used here was not maintained as inbred lines, but remaining kernels of the same cross are currently developed into inbred lines so that more seeds for future studies combining both, molecular markers and microscopy of metaphase chromosomes in pollen mother cells would be available. If we could show, that the crossing barrier in terms of multivalent formation was overcome (or not an issue at all), it would allow (the three multivalent forming chromosomes of) *S. strictum* to be used as new (secondary) rye breeding pool, what may be especially interesting in terms of disease or drought resistance. Surprisingly, in a diversity study based on molecular markers [42] a single variety Gonello (KWS Lochow), that was characterized by high frost tolerance, was related to *S. strictum* showing that this wild species may had already been used in annual rye breeding.

### 3.2. Fertility

**Figure 6:**
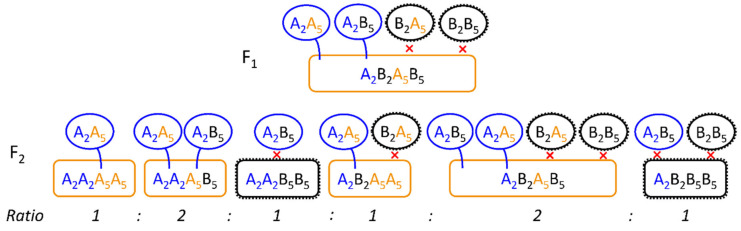
Visualization of self-fertility hypothesis for locus *S2* (Z) and *S5* (subscript 2 and 5) with the respective parental alleles for the inbred line (A) and self-incompatible genotype (B). The hypothesis displayed here was that both, pollen and stigma, carry proteins or any structure (black) on the surface that was responsible for the self-incompatibility mechanism. Those structures were expressed by a respective allele at each (multiallelic) locus. The *Z* locus was causal for the pollen (circles) and the *S* locus for the stigma (rounded rectangles). Self-fertility (*Sf*) could have been expressed by a mutation at each locus (pollen = blue, stigma = orange) that resulted in no or a special (protein) structure causing, if matched with a similar or complementary structure on the opposite surface (= two colored surfaces), successful pollination. Because the pollen was haploid and the stigma diploid, the *Z* alleles segregated 1:1 (A_2_A_2_:A_2_B_2_) and the *S5* alleles 1:2:1 (A_5_A_5_:A_5_B_5_:B_5_B_5_) and the ratio of the combination of both loci is displayed in the figure. The *Sf* allele for the stigma (*S5*) was dominant.

The locus QTL-F5 could be identical with the *S5* locus [25,26] and the high amount of explained genetic variance of this locus proved it to be the main reason for non-fertility. It was surprising, that even though we did not place isolation bags on the heads of the plants, we most probably detected a self-incompatibility locus (with a segregating self-fertility allele) to explain most of the genetic variance for fertility. The reason could be that the experiment was flowering 2–3 weeks later than the rye stand growing on the experimental station so that the main pollen cloud could not pollinate the experiment. Additionally, the genotypes were grown as single plants in a distance of each other (about 0.25 m) so that also within the experiment cross pollination was reduced. Still, for confirmation of limited self-fertility it would be necessary to use isolation bags preventing any cross-pollination in future experiments. In this study, no genotype was completely sterile. In rye, there were several loci known for self-incompatibility, three of them were located on chromosome 1R, 2R and 5R and reported most often [23,24,25,26,27] but also loci on 4R and 6R have been proposed [29]. When self-fertility is interpreted as a special allele *Sf* at those loci, we hypothesised that the expression of *Sf* lead to an universal (or complementary) structure resulting in successful pollination. However, the pollination was only successful when *Sf* was expressed in both, pollen and stigma. Important for the segregation of the respective alleles at both loci was that a pollen structure would be defined by one haplotype (n = 1x) and consequently no pollen with the “B” allele at locus *Z* could fertilize the macrogamete. This resulted in a 1:1 ratio of the “A” and “H” allele at the *Z* locus, what was proven by marker segregation. The pistil surface on the other side was in diploid stage and here a single (dominant) “A” allele must had expressed the self-fertility as we could show by the respective effect sizes (Table 2, Figure 5b). We visualized our hypothesis in Figure 6. However, we never (microscopically) studied the growth of the pollen tube on the stigma and eventually also other mechanisms could prevent the fertilization of the macrogamete.

Previous studies were mainly based on marker segregation [25,27] and in further inbreeding generations, the alleles for the *S5* locus would theoretically deviate more and more from a 1:1 ratio (of randomly segregating parental alleles), because certain genotypes could not be self-pollinated (Figure 6). In the F_3_ generation, therefore, we would expect an average ratio of the *S5* alleles of A:H:B = 3:2:1 (= 2:1 ratio of parental alleles A and B). Due to excess of pollen, the macrogametes could be successfully self-pollinated even when the *Z* locus was heterozygous so that the interaction between both loci could (principally) not be validated on marker basis. If we assume that there were only two target sides (pollen or stigma surface) affected by expressed *Sf* alleles, it may even be possible that there were more (stigma-based) self-incompatibility loci segregating in this population, that could not be detected because they were masked by the *Z* and the *S5* locus. Further, the high amount of explained genetic variation of the *S5* locus indicated that the lack of fertility was mainly dependent on self-incompatibility and not on chromosomal abnormalities and the additional QTLs we detected could also be additional self-incompatibility loci. For the identification of reasons for fertility other than self-incompatibility, additional studies of pollen vitality or germination, as done in early studies [12], could also help to better assess non-fertility. In this study, (extremely) sterile plants could also be caused by pollen sterility instead of self-incompatibility that we did not investigate. Unintentionally, the self-incompatibility loci were also selection factors for perenniality. As discussed before, the unintentional selection at the *Z* locus may have even helped to select crosses with “advanced” perennial genotypes. The second self-incompatibility locus *S5* could additionally help to shorten the distance to QTL-P5, because it explained 0.35 of the genetic covariance between non-fertility and perenniality (Table 2). Though, in following generations the most-fertile and most perennating genotypes must be selected. A random continuous self-pollination could otherwise result in a reduction of perennial genotypes.

### 3.3. Perenniality

To the best of our knowledge, this is the first mapping study for perenniality in rye. We could show, that perenniality was not caused by a single major gene like it was proposed by Dierks and Reimann-Phillip [19] and five QTLs could be located on chromosomes 2R, 3R, 4R, 5R and 7R that in combination explained 0.74 of the genetic variance. All identified QTLs were highly additive so that three of them, QTL-P4, QTL-P5 and QTL-P7, with the highest explained genetic variance, each ranging from 0.16 to 0.24 may be most interesting for breeding. However, a fixation of three QTLs simultaneously would require large population sizes and for the marker-defined clusters still a large variation between the genotypes was observed (Figure 5a). Future (backcross) populations with defined marker combinations must proof potential redundancy of the identified QTLs (genes) and whether the number of QTLs for perenniality could be reduced to still reach a high trait level. We additionally tried to estimate marker–marker interaction but concluded that it was not significant. Here, the adjustment of the genome-wide error might had been too strict. With the given method for adjustment [43] it was computationally not possible to enter all marker–marker interactions into a PCA and calculate the effective marker number from it directly. Thus, we used the number of all possible combinations of effective markers from the single marker fit as effective marker number (=q_eff_(q_eff_ − 1)) for the global-threshold adjustment of the marker–marker interactions. Additionally, the incremental fit of the fixed model effects reduced the power for the (lastly fitted) marker–marker effects and hence most probably, the procedure was generally over-adjusted. An example for epistasis could be found in rice where a dominant complementary gene action for *Rhz2* and *Rhz3* was concluded [31].

Aside from the genetic complexity, the trait was also dependent on the environment. This was shown by large genotype–location interaction (Table 1) and could be explained, by the expression of the perennial phenotype by several genes, of which each could be differently affected by environment. If we would split up the trait perenniality (and the environment) into more factors, like number of axillary buds (shoots), general number of tillers or sensitivity to vernalization, we may probably gain a better understanding of the different genetic and environmental factors. In this study, multi-environment trials were limited by the use of the F_2_ generation that we had chosen to reduce the influence of (at this point) unknown fertility reasons. With further inbreeding generations, more seeds would be available to replicate a single genotype (line) in several environments. To still not neglect this issue, we vegetatively cloned the single genotypes, resulting in plants strong enough to be tested in two (ecologically highly) different environments.

### 3.4. Species Synteny

To our knowledge, this was the first mapping study of perenniality in rye, but in other cereals perenniality has already been mapped and even further, certain loci could be connected across species (e.g., in rice, sorghum, teosinte, *Leymus*-wildrye) by comparing gene, marker or protein sequences [30,31,32,35,37,38,40,44,45]. Because rye was generally shown to be highly syntenic to some of those species [46,47], it could be interesting to compare our results with the other species. However, on a phenotypic level, the perenniality in our study differed from the other crops, because there, perennial genotypes were simultaneously characterized by rhizomatous growth. On a genomic level, it was difficult to make proper comparisons, because so far for rye only reference genome sequences for the annual crop (*S. cereale*) had been published [47,48] and functional perennation genes could principally only be found in genomes of perennial species (genotypes). If we expect similar genes for perenniality across cereals, the comparison of genomic sequences from perennation-associated QTLs in perennial species could be a valuable resource to dissect perennation from related traits and to filter the vast amount of potential gene candidates. The sequence of perennial rice, *Oryza longistaminata,* was already published [49] and perennial sorghum, *Sorghum propinquum,* was mentioned to be sequenced [45]. To our knowledge, research on perennation in the closest rye relatives, wheat (*Triticum aestivum*) and barley (*Hordeum vulgare*) ended with crosses between crop and wild relative [50,51]. In terms of basic knowledge of perenniality, studies of the model plant *Arabidopsis thaliana* or better its perennial relative *Arabis alpina* were also useful. A perenniality-related flowering gene was identified [52] and the study showed that flowers of *Arabis alpina* developed from the main shot and the axillary buds (meristems) were most important for the perennial life cycle. Another study [53] showed that the axillary buds that were developed at a distance from the shoot apical meristem (SAM) and before the onset of vernalization, remained dormant during flowering and this was the prerequisite for perenniality. Caused by vernalization and the following determination of the SAM into flowers, also axillary buds in proximity were developed and their fate of vegetative growth (in the first flowering period) was determined. This showed, that one key mechanism for perenniality was the conservation of axillary buds and consequently the isolation or even protection from plant hormones triggering flower induction and later senescence. The fact, that axillary buds were developed before flowering showed that plant resources were invested into the vegetative growth (instead of seed set) before flowering and seed yield reduction due to perenniality would mainly be caused by less (dense) tillers compared to annual plants. An important question would be, to what extent the roots were affected by the senescence of flowering shoots and how the plant can reach new nutrients, especially when plants cannot spread out by rhizomatous growth like in perennial rye. It was also shown that environmental factors like the duration of cold treatment influenced the percentage of plants that showed senescence after seed set [53].

### 3.5. Breeding Perspectives

Compared to the trait fertility, which could be reduced to a single self-incompatibility locus, perenniality remained a complex topic in which we, as a first step, identified QTLs for perenniality in rye. The trait also showed high genotype-environment interaction. In comparison to our experimental set up with intensively cherished single-plant growing practice, more farming-like practices with field plots previously showed worse results in the degree of perenniality. When perennial genotypes were grown in large-drilled yield plots, the regrowth in the second year was much lower compared to a single plant growing practice and the yield of the perennial progenies was reduced compared to annual rye sown a second time in the same plot [54] illustrating that intense intra-plot competition negatively affects perenniality. Perennial rye varieties were better suited for dual purpose use (grain + biomass), grazing or mixed cropping [3,5,54] and it is probable that such practical challenges would also appear in other perennial cereals like wheat or barley as soon as they reach higher breeding progress. In wheat, the progress was challenged by the hexaploid karyotype of wheat combined with the diploid or tetraploid karyotype of the perennial (*Thinopyrum*, *Leymus*) species [35,51]. Recombination between the genomes was lacking and as a solution amphiploid wheat was discussed to serve as potential new breeding pool [55], but has previously also been found as impractical [56]. Generally, all species comparisons highlighted that perenniality must be well defined [57] and also for rye the differences in the phenotype could be refined for more detailed studies. Especially with a focus on future breeding efforts, the identification of perenniality-related plant structures like dormant buds or shoots in the young rye plants could be a powerful selection criterion and allow the trait perenniality to be assessed even before flowering and hence to shorten the breeding cycle.

Aside from breeding perennial varieties, perenniality may be interesting as an additional tool for rye breeding. The conservation of single plants over several years without development of inbred lines would allow to implement new breeding strategies, especially for population breeding. By means of polycross or (incomplete) diallel methods [58,59] the combining ability of a single genotype (plant) could be estimated from its offspring after crossing with a tester population and based on it, superior (perennial) plants could be intercrossed after the two-year testing procedure to build up a new improved population. Such a methodology was so far only possible by special short-day cultivation practices keeping clones of the plants in a vegetative state alive over several years that was in practice not very successful [60,61]. Later on, in vitro propagation of single rye plants was tried, but after the long storage period under cold conditions the plants quickly switched into the generative stage resulting in one or a few tillers only and thus restricting seed availability. Beside this special breeding purpose, the largest potential of perennial rye was to use it as genetic resource for trait introgression into cultivated (hybrid) rye, because we observed a good resistance to leaf rust (*Puccinia recondita*) and stem rust (*P. graminis* f.sp. *secalis*).

## 4. Materials and Methods

### 4.1. Breeding Material

Breeding material of a self-incompatible perennial rye population originally derived from a cross *S. cereale* *× S. strictum* was received from Reimann-Philipp who selected for high levels of fertility and perennation over several cycles. Several plants thereof were crossed plant-wise with a self-fertile breeding line of the hybrid rye program of the University of Hohenheim (L301-N) in the greenhouse in 2014. From the resulting F_1_ plants (sown in autumn 2014), the most fertile single plant with high perenniality was self-pollinated under an isolation bag (in 2015) resulting in the F_2_ population ‘L301-N × 84/1′ that derived from a single gamete of the perennial rye population. We had to analyze this F_2_ population and could not self the population subsequently (e.g., by single-seed descent), because otherwise the non-fertile plants would have been eliminated equaling a strong (natural) selection and producing a bias to our mapping population given the correlation of fertility and perenniality.

### 4.2. Field Trials

In autumn 2015, the F_2_ population was sown in trays and transplanted in pots after having 2–3 leaves. When the plants (= genotypes) produced enough axillary shoots (tillers), 200 plants were vegetative cloned (ripped apart) resulting in four clones per genotype. Two clones of each genotype were planted as replicates in the field located in Stuttgart-Hohenheim (48°42′54″ N 9°11′22″ E, 389 m above sea level, mean annual temperature 10.1 °C, mean annual precipitation 691 mm) and two clones at ‘Oberer Lindenhof’ (48°28′26″ N 9°18′18″ E, 720 m above sea level, mean annual temperature 6.8 °C, mean annual precipitation 942 mm) close to Würtingen (St. Johann, Germany). We used spaced planting with a distance of 0.25 m from each neighboring plant. The genotypes plus some checks were grown in two randomized complete blocks at each location. The plants flowered in 2016, seeds were harvested subsequently and the plants cut by hand to 20–30 cm above the ground, so that perenniality (plant regrowth) could be assessed some weeks later according to the following.

Perenniality was assessed on a 0 to 9 scale, indicating the amount of new arising tillers in relation to the remaining stubble from the initial shoots. Zero indicated no regrowth of the plant and nine a full regrowth resulting in about the same number of tillers as remaining stubbles from harvest. To prevent false scorings due to germinated seeds that fell out by threshing, the plots were cleaned with a commercial leaf blower after harvest. In Hohenheim, the seed set or better non-seed set (“Schartigkeit”) of the heads was visually scored as percentage (0–100%) of florets that did not develop seeds (in comparison to the seed set of non-perennial self-fertile rye grown as check). Consequently, fully sterile plants were scored with 100% non-seed set and fully fertile (like standard self-fertile rye) were rated with 0% non-seed set. To simplify the wording throughout the paper, we calculated the fertility by 100% minus non-seed set and used this term instead.

### 4.3. Marker Analysis and Linkage Map Construction

From the F_2_ population, 182 genotypes and the respective parents were used for marker analysis. The DNA was extracted from about 4–5 cm long, dried leaf samples using the Macherey and Nagel (Düren, Germany) NucleoSpin 96 Plant II DNA extraction kit. Marker analysis was done with a proprietary rye 10K Infinium iSelect SNP chip at KWS SAAT SE and Co. KGaA, Grimsehlstr. 31, 37555 Einbeck, Germany. The SNPs of this assay were partially overlapping with the 5k-SNP assay of Martis et al. [46] and the 600k-SNP assay of Bauer et al. [41]. Only segregating markers were kept and coded as ABH (parent1, parent2, heterozygous). When one of the parents had a missing or heterozygous marker allele, the other parent was used as reference for ABH coding. If marker alleles were heterozygous and/or missing for both parents, the markers were dropped from the data frame. Additionally, markers with more than 10 percent missing values were dropped, resulting in 2641 markers of which 2314 were correlated with one (= redundant) in several combinations so that only a single marker thereof (with least missing values) was kept for further analysis, resulting in 789 unique markers. Those markers had 0.1% missing values on average and were imputed with the “imputeByFlanks” function from the R package ABHgenotypeR [62]. After this, still seven missing values remained in the data. Those were imputed manually. For linkage map construction we used the non-imputed data and included the redundant markers as they were located at the same position anyway, but allowed a better comparison of markers with the already published linkage map from Bauer et al. [41]. The linkage map was constructed using the R package ASmap and the “mst.map” function with “Kosambi” method [63,64]. Based on marker data, two genotypes were identified as being identical, each to a further one. The duplicates (one genotype per duplicate) were removed for linkage map construction and marker studies. For mapping, both duplicates (two genotypes per duplicate) were removed.

### 4.4. Phenotypic Analysis and Mapping Procedure

Phenotypic means were calculated by using mixed models implemented in the software ASReml for R [64,65]. Given by the experimental structure the model (1) was *y_ijkl_ =* *µ + g_i_ + l_j_ + r_jk_ + (gl)_ij_ + e_ijkl_*, with the observation *y_ijkl_* explained by the overall intercept *µ* and effects for the *i*th genotype *g*, which was modelled as fixed for best linear unbiased estimators (BLUEs) and as random to estimate variance components, and the further random effects for the *j*th location *l*, the *k*th replicate (= cloned plant) *r* nested within the location, the genotype–location interaction *(gl)_ij_* and the error term *e_ijkl_*. As the fertility was only assessed in a single location, the location effect and the respective interactions were not fitted for this trait. The heritability was calculated by *H^2^ =* *σ_g_^2^/(σ_g_^2^* *+ av.VD/2)*, where *σ_g_^2^* is the genetic variance and *av.VD* the mean variance of a difference of two BLUEs [66].

The QTL-mapping procedure was based on single marker regression fitting each marker as codominant (first) and as dominant (second) fixed effect. The codominant (cd) markers were coded as 0, 1, 2 (A, H, B) and the dominant (d) as 0, 1, 0. For the trait perenniality, also random effects for the (cd and d) marker-location interactions were fitted. For each (fixed) effect the *p*-value was extracted from a Wald-test statistic of the fixed effects. Additionally, a *p*-value for the combination of both effects was calculated by adding up the (incremental) Wald-statistics of both effects and calculating *p*-values based on Chi-square statistics with and adjusted number of degrees of freedom (df = 2). The global significance threshold was calculated by the simpleM method [43]. Comparable with Bonferroni correction, the defined genome-wide significance threshold α = 0.05 was divided by the number of tests (markers) *q* in order to obtain the SNP-wise significance level. Due to correlation of the markers, the tests were not independent and α was divided by an effective number of markers q_eff_ instead. The effective number of markers q_eff_ was calculated by running a principal component analysis (PCA) based on the marker data and the number of eigenvalues that explained 99.5% of the variation of the SNP data. PCA was based on a correlation matrix of the markers coded in 0, 1, 2 (A, H, B) and calculated by using the “eigen” function in R [64]. The procedure was initially developed for genome-ide association studies (GWAS) [43] and we considered the linkage mapping as special case of GWAS resulting in an even smaller number q_eff_ due to the high linkage of markers, compared to GWAS studies.

To adjust for masking effects of genes located on other chromosomes than the single marker in the fit, we additionally run two scans with models including markers (cd and d) as fixed cofactors fitted in the model sequence before the actual marker under testing (cofactor-based mapping, CM). The two additional scans differed in terms of the cofactor selection. For the first additional scan CM1, from each chromosome the most significant markers were chosen, that passed the global significance threshold in a cofactor-free scan (compare [67]). For the second additional scan (CM2), a simultaneous forward- and backward-selection procedure was used to identify the respective cofactors. For simplicity reasons and computational speed, this procedure was based on a linear model including only the BLUEs and markers in cd coding. The procedure was implemented as “steps” function in R [64]. We started the procedure with a Null model (only intercept) and used the (Schwarz) Bayesian information criterion (BIC) [68] by setting k = log(N_genotypes_). Similar to the single marker regression without cofactors, *p*-values for cd and d marker effects were extracted from the Wald-test statistics.

For all markers identified as significant we calculated effect sizes plus standard errors from the estimated coefficients and coefficient error variance. The explained genetic variance p_G_ was calculated as reduction in the genetic variance estimated in the phenotypic model (1) by additional marker effects, divided by the full genetic variance estimated in model (1). We additionally ran a bivariate model including both, perenniality and non-fertility in one model. We fitted random effects with unstructured variance-covariance matrix for the genotype and the residual and with diagonal variance-covariance matrix for the replicate. Only data with records from both traits was used and thus no location effect was fitted. Similar to p_G_, the explained genetic covariance p_CovG_ was calculated as reduction in the genetic covariance estimated in the bivariate phenotypic model by additional marker effects divided by the full genetic covariance estimated in in the bivariate phenotypic model.

To detect potential epistasis of marker1 (m1) with marker2 (m2), a scan with all marker–marker interactions and possible d and cd combinations was run. The (incremental) sequence of fixed effects was the following: m1_cd_ + m2_cd_ + m1_d_ + m2_d_ + m1_cd_:m2_cd_ + m1_d_:m2_d_+ m1_d_:m2_cd_. The m1_d_:m2_cd_ interaction was tested also vice versa (m2_d_:m1_cd_). No additional cofactors were used in this run, but for the trait perenniality all fixed marker effects were additionally fitted as random marker-location interactions. We defined the q_eff_ for global significance threshold of epistasis effects as product of q_eff_ and (q_eff_-1) as this would be the number of all possible combinations. Due to the high number of marker–marker combinations tested (n = 621,732) we did not run a PCA and not deduced q_eff_ specifically for the interactions.

## 5. Conclusions

We could show that perenniality in rye was quantitatively inherited and we identified five QTLs with medium effect sizes of which some acted dominantly. This quantitative nature could be challenging for breeding of perennial rye and if breeders aim for perennial varieties, future studies should investigate the impact of the environment and to what extent selection on perenniality-related plant structures or with molecular markers could shorten breeding cycles. Reduced fertility was related to cytogenetic causes in previous studies. We could show, that in our material it was mainly related to self-incompatibility by identifying a locus that explained 0.64 of the genetic variance and is most probably the *S5* locus, known from other studies. If the self-incompatibility is considered consequently, reduced fertility should not be a concern in future breeding programs.

## Figures and Tables

**Table 1:** Variance components (Var.comp.) with standard errors (St. Error) and entry-mean heritability (H^2^) for the traits perenniality (scored in 1-9 scale) and fertility (scored from 0 to 100%). Fertility was assessed in one location and hence some factors could not be assessed (n.a.).

	Perenniality (1–9)		Fertility (0–100%)	
	Var. Comp.	St. Error	Var. Comp.	St. Error
Location (L)	0.22	0.39	n.a.	n.a.
Replicate	0.08	0.09	2.6	4.4
Genotype (G)	3.72	0.51	313.7	38.9
G×L	1.18	0.21	n.a.	n.a.
Residual	1.39	0.10	94.9	9.5
H^2^	0.81		0.87	

**Table 2:** QTL mapping results for the traits “perenniality” (P) and “non-fertility” (F). For each locus (QTL) the markers with the smallest *p*-values are reported. Two markers for a locus are reported when different methods (single marker regression without cofactors and with two different sets of cofactors) resulted in a different marker with smallest *p*-value, but an overlapping locus.

Name	Marker	Chr.	Pos.	CI L	CI R	Pos. [41]	Pval c	Pval d	Pval cd+d	Eff. cd	SE cd	Eff. d	SE d	n_A_	n_B_	n_H_	p_G_	p_CovG_
Perenniality																		
QTL-P2	Contig1964 ^a^	2	121.0	79.2 ^a^	123.3 ^a^	153.6 *	6.4 × 10^−1^	1.7 × 10^−4^	3.6 × 10^−5^	−0.69	0.42	1.32	0.30	62	18	96	0.07	0.00
QTL-P3	isotig21327 ^b,c^	3	71.1	66.9 ^c^	73.3 ^c^	100.5	1.0 × 10^−2^	2.9 × 10^−1^	3.0 × 10^−1^	0.58	0.19	0.34	0.27	45	44	87	0.04	−0.08
QTL-P4	C9941_1700 ^a^	4	72.0	70.3 ^a^	75.4 ^a^	89.0 *	5.0 × 10^−8^	8.9 × 10^−1^	6.4 × 10^−7^	1.15	0.18	0.07	0.45	51	40	85	0.16	0.32
	Contig1605 ^b,c^	4	74.6	72.0 ^c^	75.4 ^c^	94.7 *	1.5 × 10^−7^	7.6 × 10^−1^	5.2 × 10^−6^	1.11	0.18	0.14	0.39	51	41	84	0.16	0.33
QTL-P5	C14811_2522 ^a,b,c^	5	77.0	71.9 ^a^	80.8 ^a^	88.8	8.6 × 10^−11^	9.2 × 10^−1^	6.4 × 10^−8^	1.34	0.17	−0.04	0.29	40	49	87	0.23	0.39
QTL-P7	isotig17332 ^a,b,c^	7	0.6	0.0 ^a^	2.3 ^a^	1.2 *	3.4 × 10^−4^	5.5 × 10^−7^	4.8 × 10^−8^	0.64	0.18	1.61	0.27	50	36	90	0.24	0.09
Contig1964 + isotig21327 + C9941_1700 + C14811_2522 + isotig17332																	0.74	0.80
**Fertility**																		
QTL-F1a	isotig11337 ^b^	1	29.7	27.2 ^b^	30.6 ^b^	62.7 *	9.1 × 10^−4^	2.0 × 10^−1^	3.4 × 10^−4^	−7.2	0.23	3.6	0.30	37	36	103	0.06	0.14
	isotig22259 ^c^	1	33.9	30.6 ^c^	34.8 ^c^	68.3 *	1.3 × 10^−2^	1.4 × 10^−1^	2.5 × 10^−3^	−5.8	0.24	4.2	0.30	37	32	107	0.04	0.11
QTL-F1b	Contig1017 ^a^	1	63.2	59.3 ^a^	66.6 ^a^	91.6 *	1.7 × 10^−4^	3.0 × 10^−3^	3.2 × 10^−6^	−7.7	0.22	8.1	0.29	37	38	101	0.12	0.17
QTL-F4	isotig03456 ^a^	4	78.2	69.2 ^a^	87.7 ^a^	103.1 *	2.7 × 10^−5^	7.7 × 10^−3^	1.7 × 10^−6^	−7.3	0.20	−7.2	0.29	53	38	85	0.13	0.29
	Contig1437 ^b,c^	4	86.2	83.3 ^c^	87.1 ^c^	126.5 *	1.6 × 10^−5^	2.4 × 10^−1^	1.3 × 10^−5^	−7.9	0.20	−3.2	0.29	37	41	82	0.11	0.25
QTL-F5 (*S5*)	C28789_183 ^a,b,c^	5	41.7	40.6 ^a^	42.0 ^a^	44.9 *	2.8 × 10^−37^	5.5 × 10^−15^	9.5 × 10^−33^	−18.4	0.14	14.9	0.20	48	38	90	0.64	0.35
isotig11337 + Contig1017 + isotig03456 + C28789_183																	0.77	0.71

The letter superscripts indicate in which model the respective marker passed the threshold and was the most significant: ^a^ single marker regression without cofactors, ^b^ single marker regression with marker cofactors selected from a first run and ^c^ single marker regression with marker cofactors selected by information criterion based cofactor selection. The superscript also indicates the method used for confidence interval estimation. For each marker the following parameters are reported: the chromosome (Chr.) and position (Pos.) based on a population specific linkage map, based on the linkage map published by [41] where * indicates that this marker could not be found on the public map and the position from the most closely linked marker was taken instead, confidence limits (CI left and CI right) based on log_10_(*p*-value) drop in 1, *p*-values (Pval) for codominant (c) effects, dominant (d) effects and the combination thereof (cd + d) as well as the effect sizes (Effect) with respective standard errors (SE), the counts (n) for the ABH-marker alleles, the explained genetic variance (p_G_), and the explained genetic covariance (p_CovG_). For estimation of the respective parameters, each marker was used as fixed effect (cd + d) in a separate model and only for calculation of total explained variance a model was fitted combining cd + d effects of all markers simultaneously.

## Data Availability

The SNP marker chip we used in this study was proprietary to KWS SAAT SE and Co. KGaA and thus the marker data is not publicly available. Marker sequences for the most important markers can be found in the Supplementary Table S1 and were additionally referenced on a previously published linkage map [41]. Seeds for perennial rye genotypes can be requested from the corresponding author. The material used in this study was not maintained.

## Supplementary Materials

The following are available online at https://www.mdpi.com/article/10.3390/plants10061210/s1, Table S1 DNA sequences of the reported markers; Figure S1 Pearson correlation of markers; Figure S2 LOD scores for mapping the phenotype perenniality by single marker regression; Figure S3 LOD scores for mapping the phenotype perenniality by single marker regression and using additional markers from cofactor selection as cofactors; Figure S4 LOD scores for mapping the phenotype perenniality by single marker regression and using additional markers from a first run as cofactors; Figure S5 LOD scores for mapping the phenotype fertility by single marker regression; Figure S6 LOD scores for mapping the phenotype fertility by single marker regression and using additional markers from cofactor selection as cofactors; Figure S7 LOD scores for mapping the phenotype fertility by single marker regression and using additional markers from a first fit as cofactors; Figure S8 Genotype means for the trait perenniality clustered by marker alleles; Figure S9 Display of codominant-codominant marker–marker interaction of mapping perenniality; Figure S10 Display of dominant-dominant marker–marker interaction of mapping perenniality; Figure S11 Display of codominant-dominant marker–marker interaction of mapping perenniality; Figure S12 Display of codominant-codominant marker–marker interaction of mapping fertility; Figure S13 Display of dominant-dominant marker–marker interaction of mapping fertility; Figure S14 Display of codominant-dominant marker–marker interaction of mapping fertility.
