# Supplementary material for: Perennial Rye: Genetics of Perenniality and Limited Fertility
Source: Plants (Basel). 2021 Jun 14;10(6):1210. doi: 10.3390/plants10061210 (PMC8232189; doi:10.3390/plants10061210)
Supplement: Supplementary file 1 [file plants-10-01210-s001.zip › plants-1231531-supplementary.pdf]

plants

MDPI

Article

Perennial rye: genetics of perenniality and limited fertility

Paul Gruner and Thomas Miedaner *

State Plant Breeding Institute, University of Hohenheim, 70593 Stuttgart, Germany; Paul.Gruner@uni-hohenheim.de

* Correspondence: miedaner@uni-hohenheim.de; Tel.: +49-711-45922690

Supplementary Materials

Table S1 Flanking DNA sequences of the 10K-Infinium iSelect SNP chip for the markers reported in this paper.

SNP-Name	Flanking sequence and polymorphic bases [in brackets]
Contig1964	CTGCAAGGCTAGCTTCACAAGCACATTCAGATG[A/G]CCTGGTGCACAAAATAGTATGTAACAGGGCAGCACATAATGACAAAATATACATGGGAGCGAGGTCAATATATACCATGGCCTACTATGCCATATATTCT
isotig21327	CATGGGTGGGTTCAACCGTCTTCTGGGTTGGAGAATGGTTTATAAAGAAAATGCCATTTGTAAAGCACATATATTCAGCATCAAAGCAAGTCAGCACTGC[C/T]ATTTCACCAGATCAAAATACGACAGCATTTAAAGAAGTTGCAATTATTCGGCATCCRCGTGTTGGTGAATATGCATTCGGATTTATAACATCAACTGTTG
C9941_1700	GCTGCAATCCCTCCTGAAAKGTGAAAACAGAAAGCACATCATGCTCCTCCCATCGGCCC[G/T]CGGAAACAACTGTCGAAATGGGCTAGAGTTCATAGAAGTTATGAGAAAACTAAAATCCGA
Contig1605	CAGGCTGCTTTTTAAAAYTGAAGGTGATCTTCAAAGTTGCACTCTGGAAAGCTGTGATTGATGGGTAAGCTGCATTCGCATCAGGAAGTGGTGTACTTGG[A/G]GACAAACCATCGAGAAGATCTATAGCTTTGAGGTGTTCCAGGAGCATGTTTAGTCACTTGGCCAGGAGAGCTTATGTTTGATGTTGCCGGCGGACCATTT
C14811_2522	ACGCCATGCGCATCTTGTTTGAATCCGTGGCACCCYTTTTAGTATATTTGCGTGAGGA[C/T]TGGATAAGCTTATGTCTTGCAAACGTATGCCGCCCGAACTACTGTCATGTTCGTGTAGAT
isotig17332	TCCAGGAAATTGCTGGTGGTCTTGATGGTGCTCTTACAGCCACAATTAGCCCTGTCGGAGTGAATGAGAACCAAATGAAGCAGAAGACATTATTGTTGAA[A/G]CAAAGTATTAAGCTAYTAGAAAGTTTGCAGTCCTGCTGGAGTGATGATGTTCTTGTTTTCTCTCACTCTGATAAATTCCTCMGATTGTCCTTGCAGCTTA
isotig11337	TCTAATTATTTAATTCCTACAGTAAATAAAAGGTAAAACATCCCGGGCGTTCGTCGTCAGCCAGCTACCAAGGTTACAGGGACAGTTTCTTTGTTTTTCC[C/T]TTTTGATTGAAACTTCAGACCAGACAAAGCTTGGTGAAGTTTGCGAATGTCCAATTGATGGGACATGTCCTGTGGAGTGGCATCTAATTTGATCCATAAT
isotig22259	CCTGGACACCCAAAACATTTGCGGATATAACTGCYGAAAGTTTATCAATCTTCAAAGTTGTGCACCCAATCCCAGAGATTCTAATTCTTGGTTGCGGGAA[A/G]TACGTCCAGCCAGTCAGTCCTGAGCTGCGCAAGTTCATTCGRTCAACWGGGATGAAACTGGAGGCCATCGATTCGAGGAATGCGGCATCGACCTATAATA
Contig1017	TGATGAACTTGGAATCAAGAATTGTGGCATCTGAAGATATTGGAAGACAGGTTCTGACTTATGGGCAAAGGAAGCCCGTCGAGCACTTCGTGAAAGCTGT[C/T]GAGGCGACCACCCTGAACGACATCTCTATGGTTGCACAGAAGATAATCTCTTCACCGCTTACATTGGCATCATGGGGCGATGTTATCCACGTCCCGAGCT
isotig03456	CCGCCTCGTATCATATCCTCTGCKCTTTTGCGCTTTTGCTCGCACGAAGCGCATCGCCAGAAGCCCACTGATAGAACGAGCAAGCATCCATCCACCMGGC[A/G]ATCTGACCTCGGAATCACTTCTCATCAGGCAGGCACACTTGCAGAATATTTCCGACTGATGGAATGGCACGGTATGGAGAATTGGGCATCTGCGGCGGCT
Contig1437	TAATGGCCAAGTTATTACTTGAGAAATTCGAGGAGAAATGGCTCCAGCTTCTCCCTAAAGTTGAGAATGAGGAAAGGAAACAACAGGTGGAAYCAAACGA[G/T]GCTCCAACCACAGACACTTCTCCGGAAGATGCTATTGCACAATTAGCAAAAGAKACTGATGATGAGCTGAATGAGATTAATAAGCAGCTGGAGCWGCTCC
C28789_183	GAGCTTTGGAGTGTTATGAGAAAGAAGCCTACTCACATAATAAAAAATGCCCATCCAGC[A/G]TTAGCTCCTAGTTCACTTGACAGTGCTGATGAAAAGTTGCCCAAAGAAAATGGAATTTGT

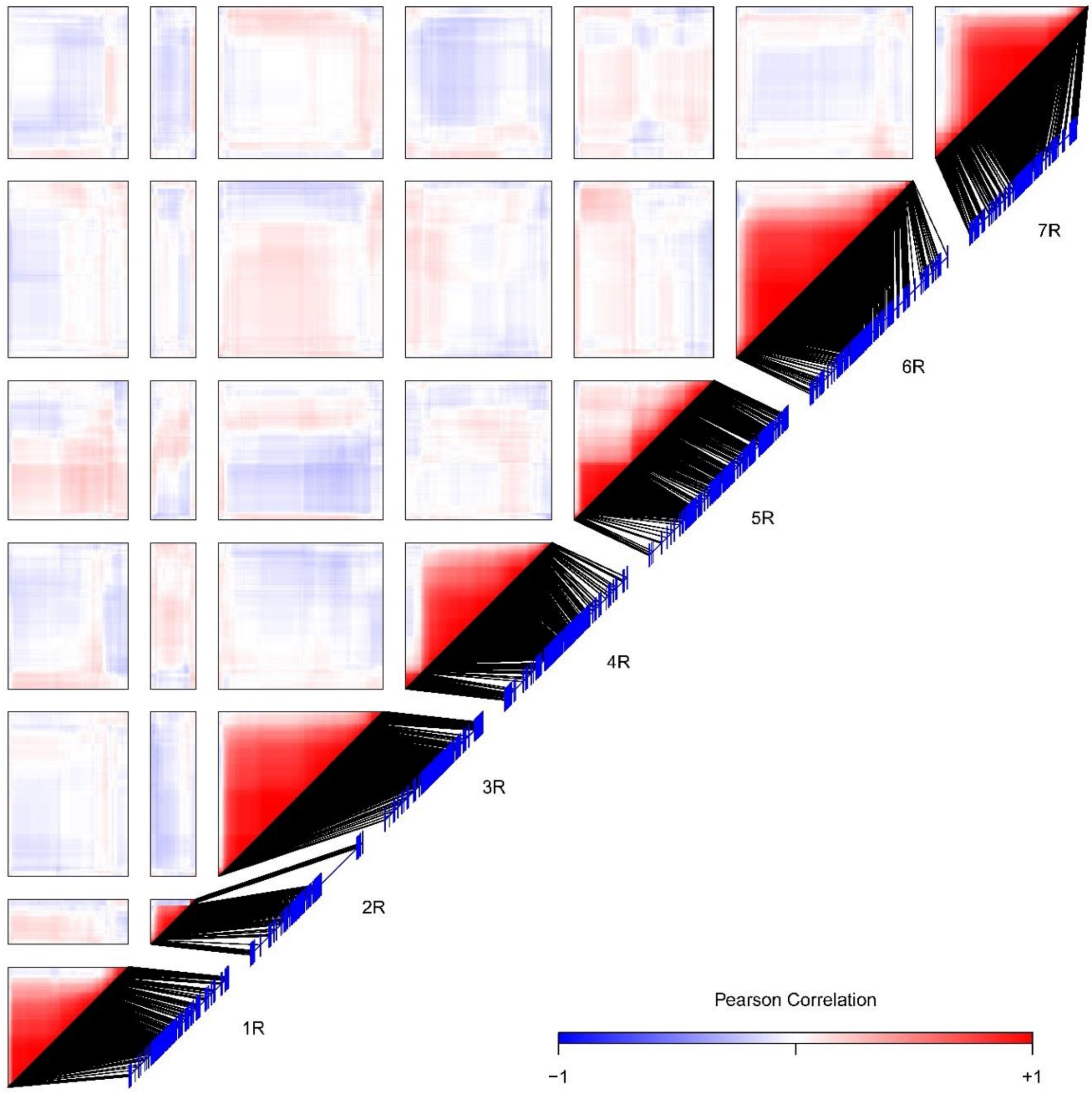

Figure S1 Pearson correlation of markers coded 0,1,2 (A,H,B) displayed as color (legend bottomright). The markers from the different linkage groups (1R-7R) are separated by boxes. The markers of the equally scaled marker-marker matrices are connected with the position on the linkage map (on the diagonal) constructed from the same marker data. Each blue vertical line is a single marker.

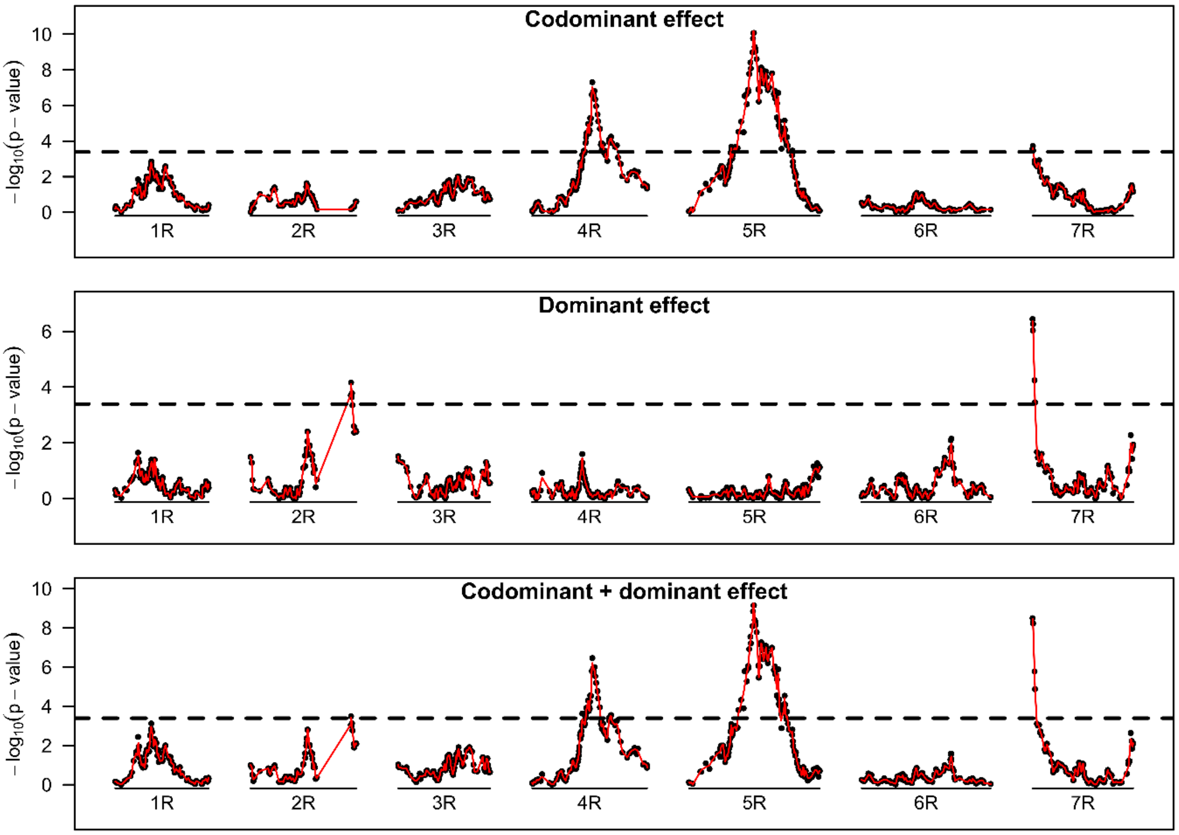

Figure S2 LOD-scores (= -log₁₀(p-value)) for mapping the phenotype perenniality (1-9) by single marker regression. All markers were fitted as linear (fixed) effects simultaneously and in the sequence of first codominant (0,1,2) and second dominant (0,1,0) coding. The p-values for the single effects were calculated by a Wald-test and the combination by adding up the Wald test statistics of the single effects and comparing with a Chi-squared distribution with two degrees of freedom. The full-model included also a (codominant and dominant) marker-location interaction for the main marker.

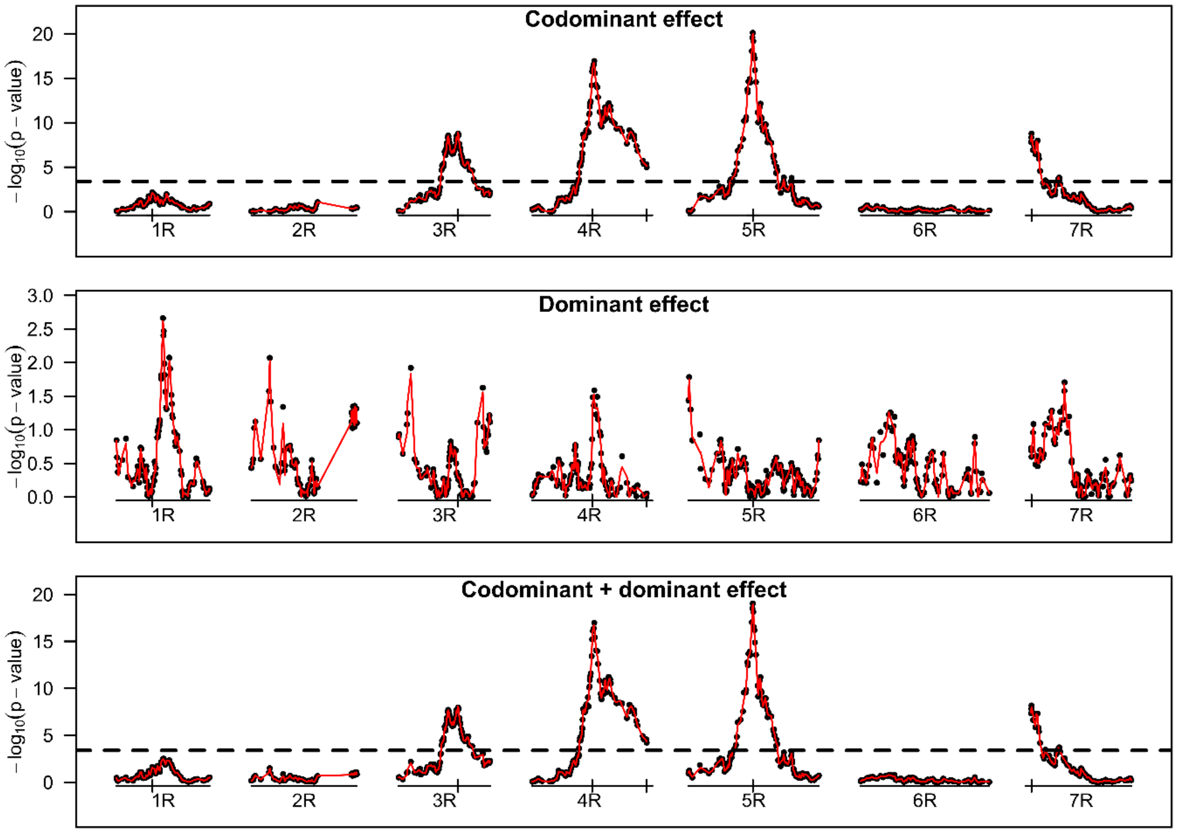

Figure S3 LOD-scores (= -log10(p-value)) for mapping the phenotype perenniality (1-9) by single marker regression and using additional markers as cofactors (ticks on chromosome axis). All markers were fitted as linear (fixed) effects simultaneously and in the sequence of first codominant (0,1,2) and second dominant (0,1,0) coding. The cofactors were selected by the a linear model with best linear unbiased estimators and markers and step()-procedure with simultaneous forward- and backward- selection based on the Bayesian information criterion implemented in R (R Core Team 2019). all markers from the same chromosome as the main effect were excluded as cofactors. The cofactors were fitted first followed by the main marker effects of interest. The p-values for the single effects were calculated by a Wald-test and the combination by adding up the Wald test statistics of the single effects and comparing with a Chi-squared distribution with two degrees of freedom. The full-model included also a (codominant and dominant) marker-location interaction for the main marker. For the marker cofactors, no marker-location interaction was fitted.

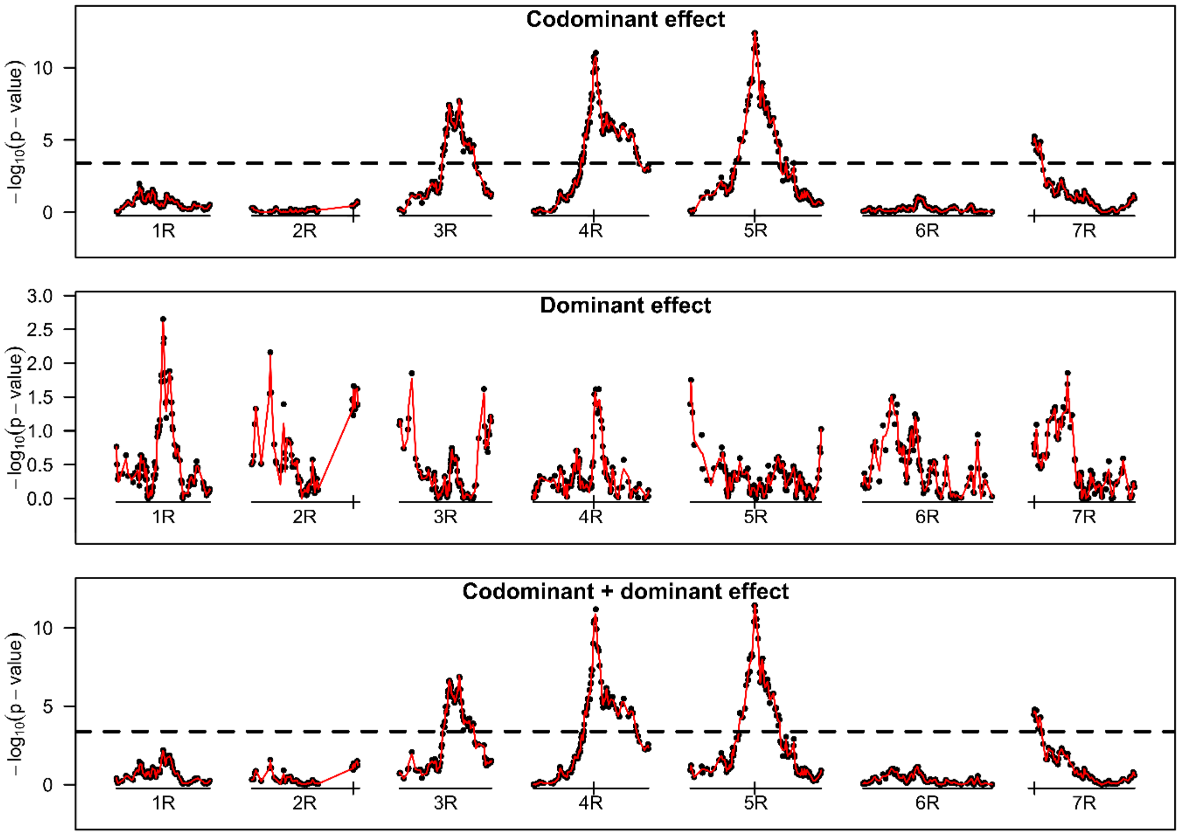

Figure S4 LOD-scores (= -log10(p-value)) for mapping the phenotype perenniality (1-9) by single marker regression and using additional markers as cofactors (ticks on chromosome axis). All markers were fitted as linear (fixed) effects simultaneously and in the sequence of first codominant (0,1,2) and second dominant (0,1,0) coding. The cofactors were selected based on a previous run of single marker regression and the lowest p-value per chromosome. In the final testing procedure all markers from the same chromosome as the main effect were excluded as cofactors. The cofactors were fitted first followed by the main marker effects of interest. The p-values for the single effects were calculated by a Wald-test and the combination by adding up the Wald test statistics of the single effects and comparing with a Chi-squared distribution with two degrees of freedom. The full-model included also a (codominant and dominant) marker-location interaction for the main marker. For the marker cofactors, no marker-location interaction was fitted.

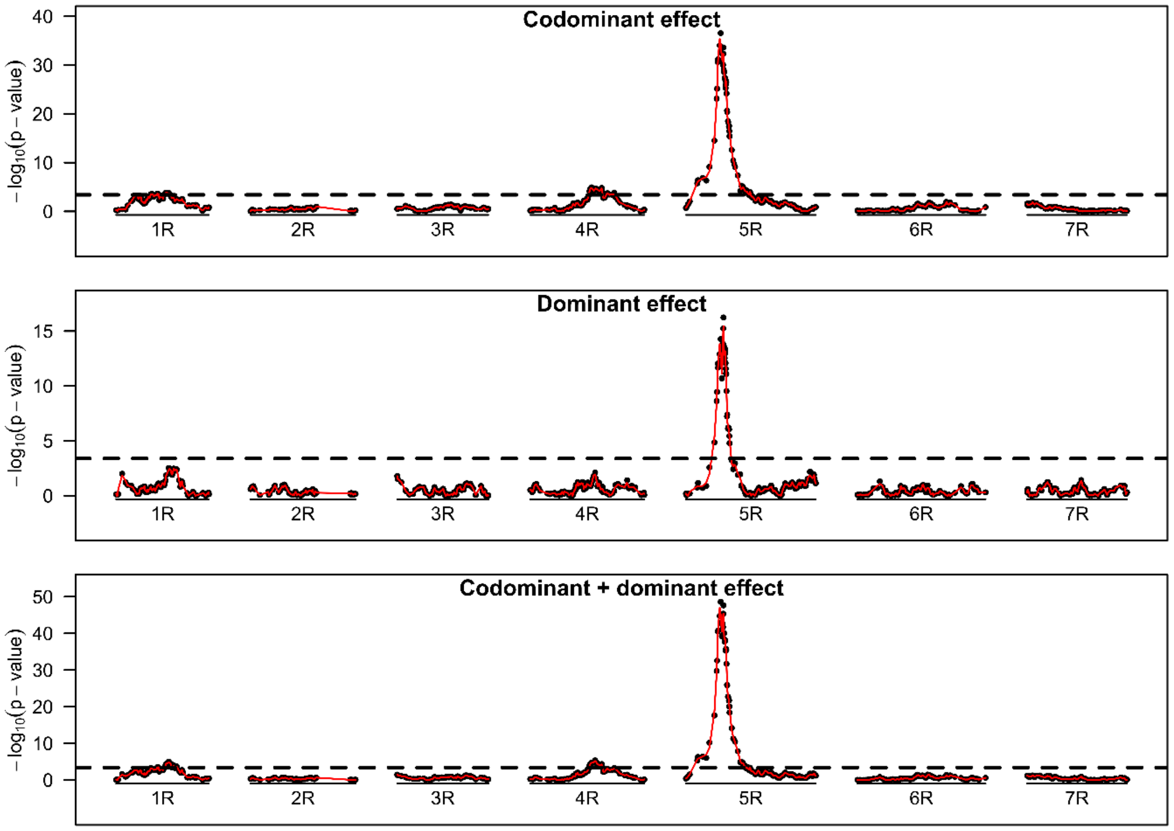

Figure S5 LOD-scores (= -log10(p-value)) for mapping the phenotype fertility (0-100%) by single marker regression. All markers were fitted as linear (fixed) effects simultaneously and in the sequence of first codominant (0,1,2) and second dominant (0,1,0) coding. The p-values for the single effects were calculated by a Wald-test and the combination by adding up the Wald test statistics of the single effects and comparing with a Chi-squared distribution with two degrees of freedom.

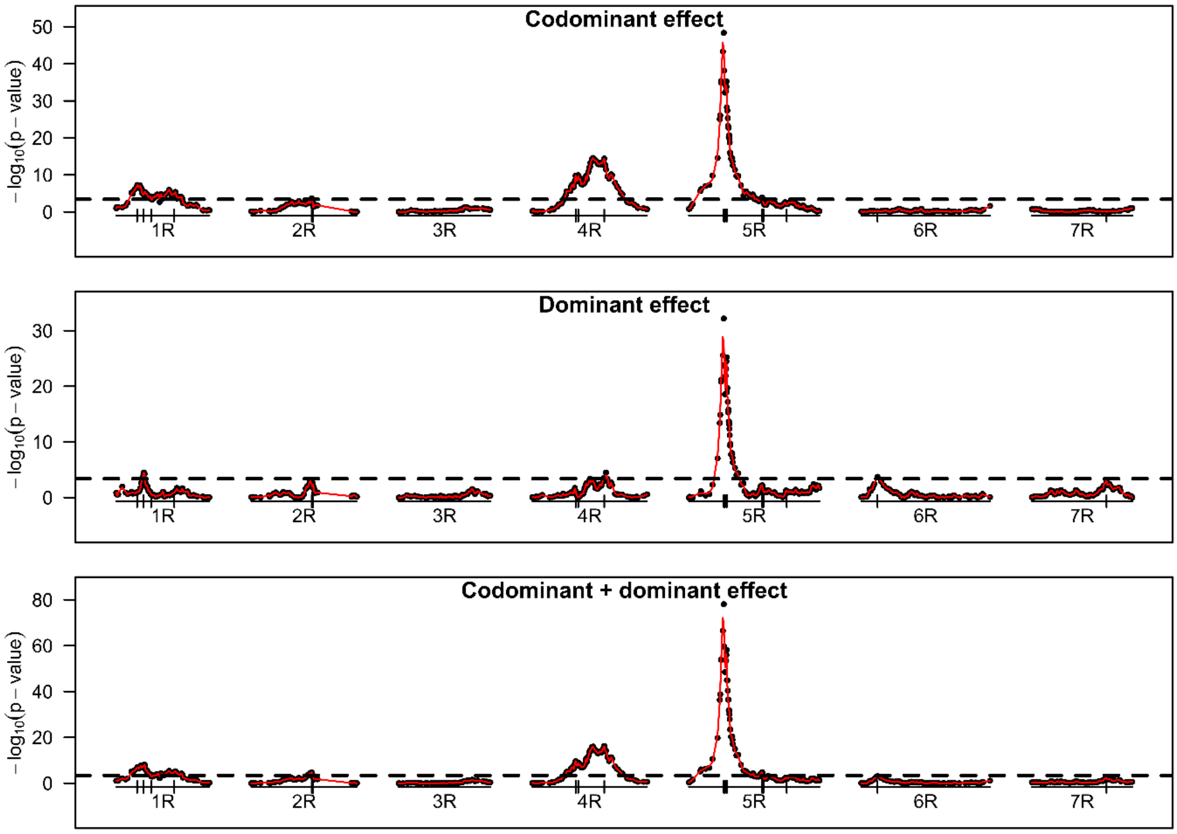

Figure S6 LOD-scores (= -log10(p-value)) for mapping the phenotype fertility (0-100%) by single marker regression and using additional markers as cofactors (ticks on chromosome axis). All markers were fitted as linear (fixed) effects simultaneously and in the sequence of first codominant (0,1,2) and second dominant (0,1,0) coding. The cofactors were selected based on a previous run of single marker regression and the lowest p-value per chromosome. In the final testing procedure, all markers from the same chromosome as the main effect were excluded as cofactors. The cofactors were fitted first followed by the main marker effects of interest. The p-values for the single effects were calculated by a Wald-test and the combination by adding up the Wald test statistics of the single effects and comparing with a Chi-squared distribution with two degrees of freedom. The full-model included also a (codominant and dominant) marker-location interaction for the main marker. For the marker cofactors, no marker-location interaction was fitted.

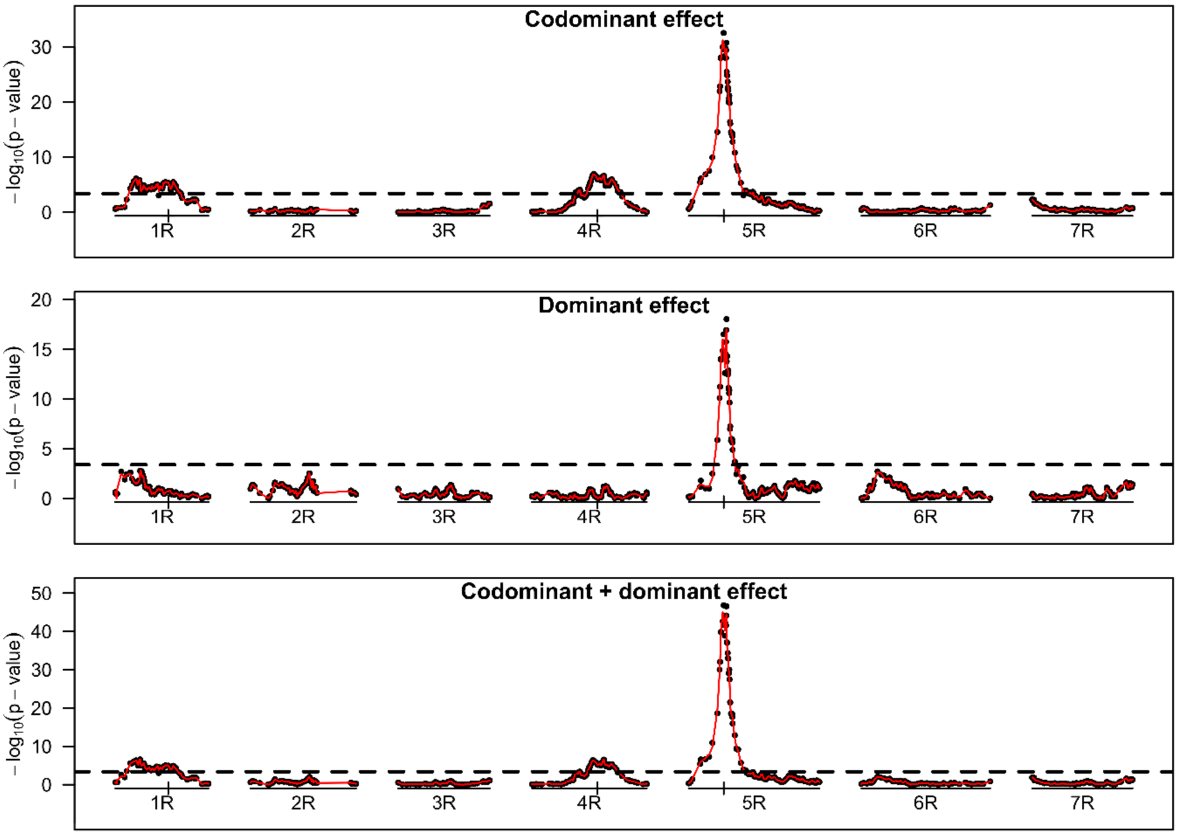

Figure S7 LOD-scores (= -log₁₀(p-value)) for mapping the phenotype fertility (0-100%) by single marker regression and using additional markers as cofactors (ticks on chromosome axis). All markers were fitted as linear (fixed) effects simultaneously and in the sequence of first codominant (0,1,2) and second dominant (0,1,0) coding. The cofactors were selected based on a previous run of single marker regression and the lowest p-value per chromosome. In the final testing procedure, all markers from the same chromosome as the main effect were excluded as cofactors. The cofactors were fitted first followed by the main marker effects of interest. The p-values for the single effects were calculated by a Wald-test and the combination by adding up the Wald test statistics of the single effects and comparing with a Chi-squared distribution with two degrees of freedom. The full-model included also a (codominant and dominant) marker-location interaction for the main marker. For the marker cofactors, no marker-location interaction was fitted.

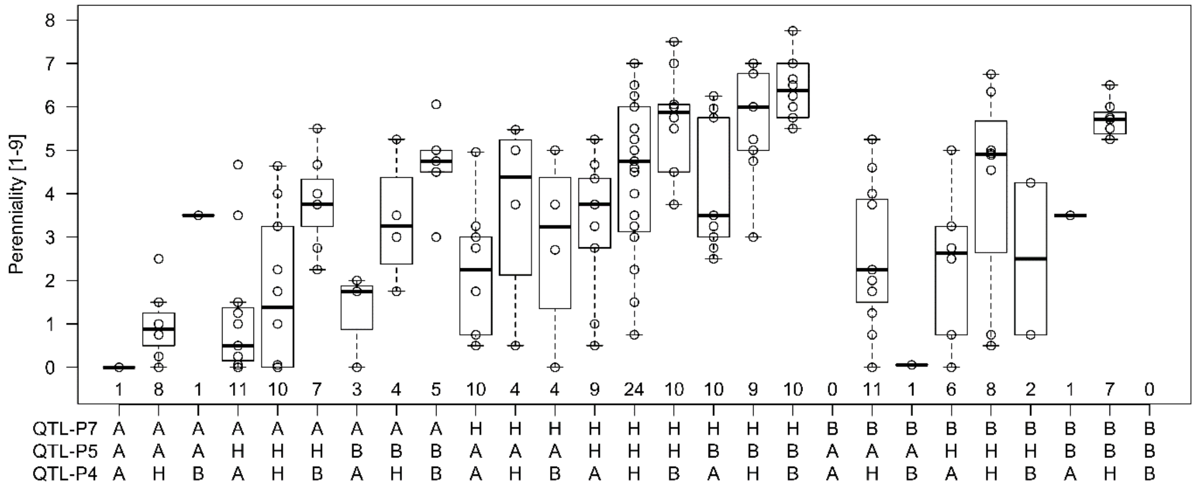

Figure S8 Genotype means for the trait perenniality clustered by marker alleles A, H, B (x-axis) of the three QTLs (QTL-P4, QTL-P5, QTL-P6) explaining the most genetic variance. Genotype means were additionally plotted as dots in the in the boxplot display (box at first and third quartile with median in center). The number of genotypes with the respective marker allele (combinations) is written above the x-axis within the plot.

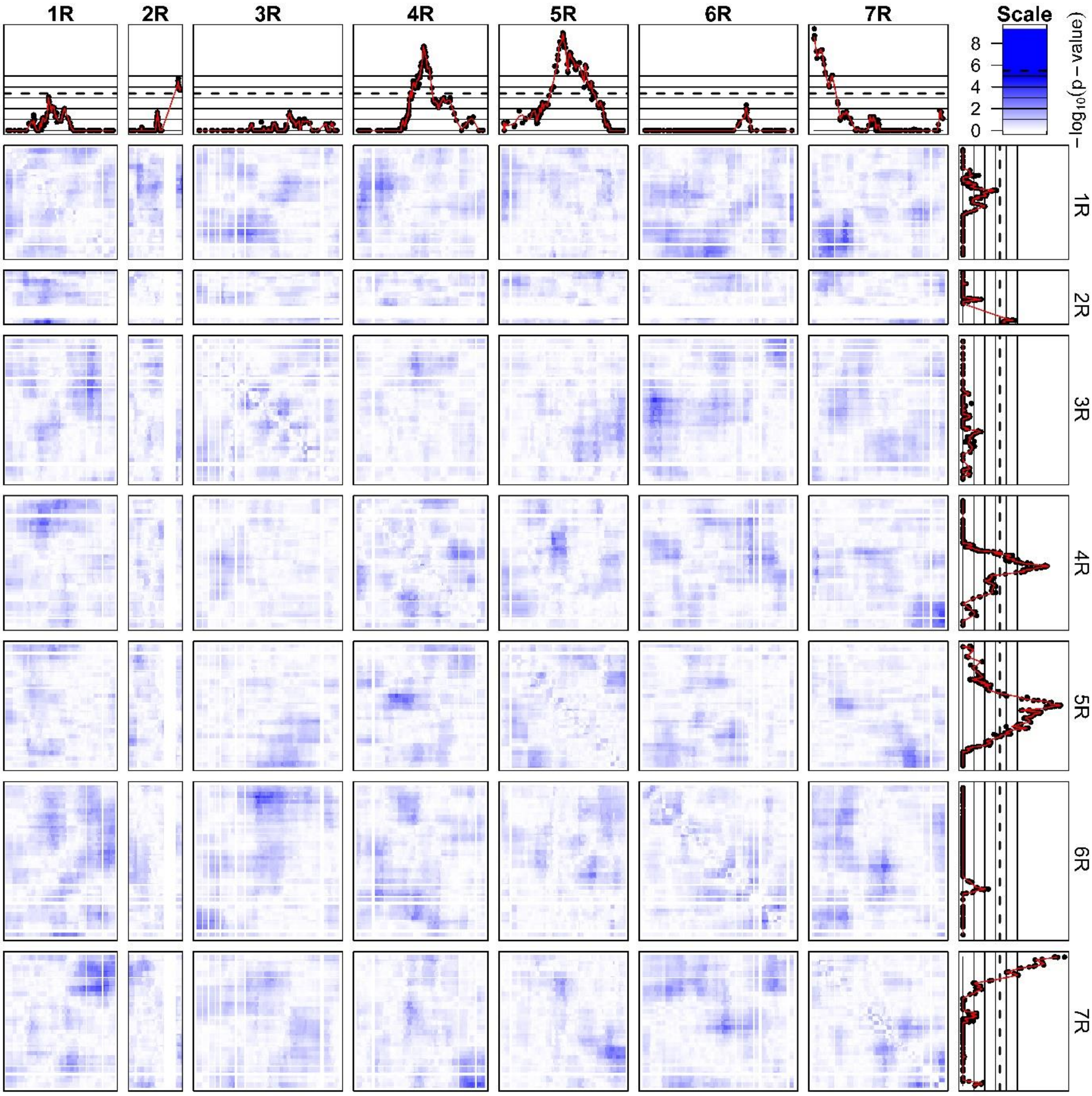

Figure S9 Display of codominant-codominant marker-marker interaction of mapping perenniality. Each box with shades of blue corresponds to a chromosome-chromosome combination and the color scale with the respective LOD score (-log10(p-value)) is shown in the upper left corner. This is legend box shows also the axis scale for the LOD-curves of the single marker fits (codominant + dominant combined without cofactors). The display is symmetric, so that comparisons in both directions result can be made. Continuous horizontal lines are plotted for better visualization in LOD curves and color legend. Dashed lines are plotted at the level of the global significance threshold. The estimation of the marker-marker interactions was based on a model with fixed marker effects in the following sequence: m1_codominant + m1_dominant + m2_codominant + m2_dominant + m1_codominant: m2_codominant + m1_dominant: m2_dominant + + m1_dominant: m2_codominant + m1_codominant: m2_dominant, where codominant markers were coded 0,1,2 (A,H,B) and dominant markers were coded 0,1,0 (A,H,B).

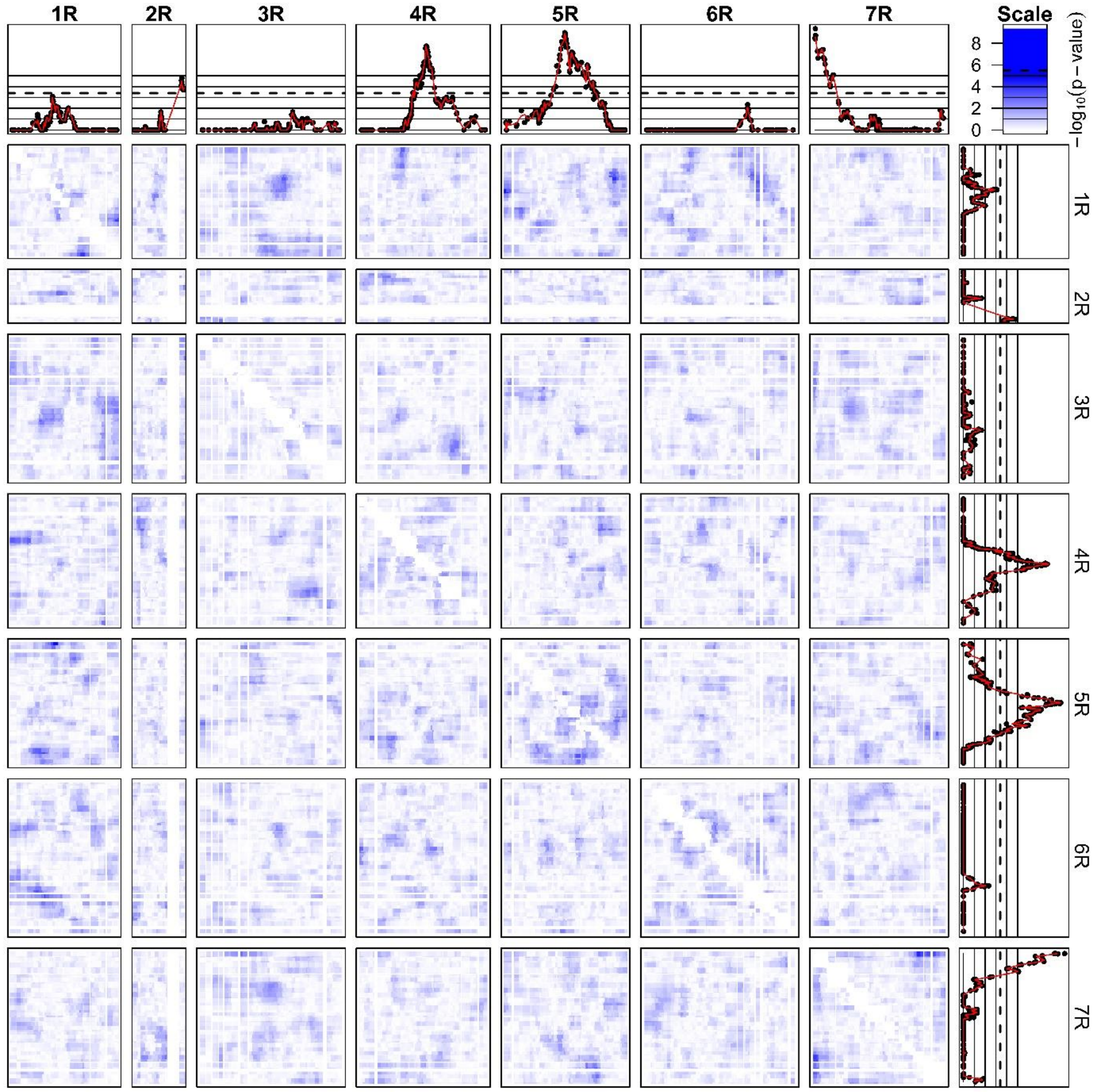

Figure S10 Display of dominant-dominant marker-marker interaction of mapping perenniality. Each box with shades of blue corresponds to a chromosome-chromosome combination and the color scale with the respective LOD score (-log$_{10}$(p-value)) is shown in the upper right corner. This is legend box shows also the axis scale for the LOD-curves of the single marker fits (codominant + dominant combined without cofactors). The display is symmetric, so that comparisons in both directions result can be made. Continuous horizontal lines are plotted for better visualization in LOD curves and color legend. Dashed lines are plotted at the level of the global significance threshold. The estimation of the marker-marker interactions was based on a model with fixed marker effects in the following sequence: m1_codominant + m1_dominant + m2_codominant + m2_dominant + m1_codominant: m2_codominant + m1_dominant: m2_dominant + m1_dominant: m2_codominant, where codominant markers were coded 0,1,2 (A,H,B) and dominant markers were coded 0,1,0 (A,H,B).

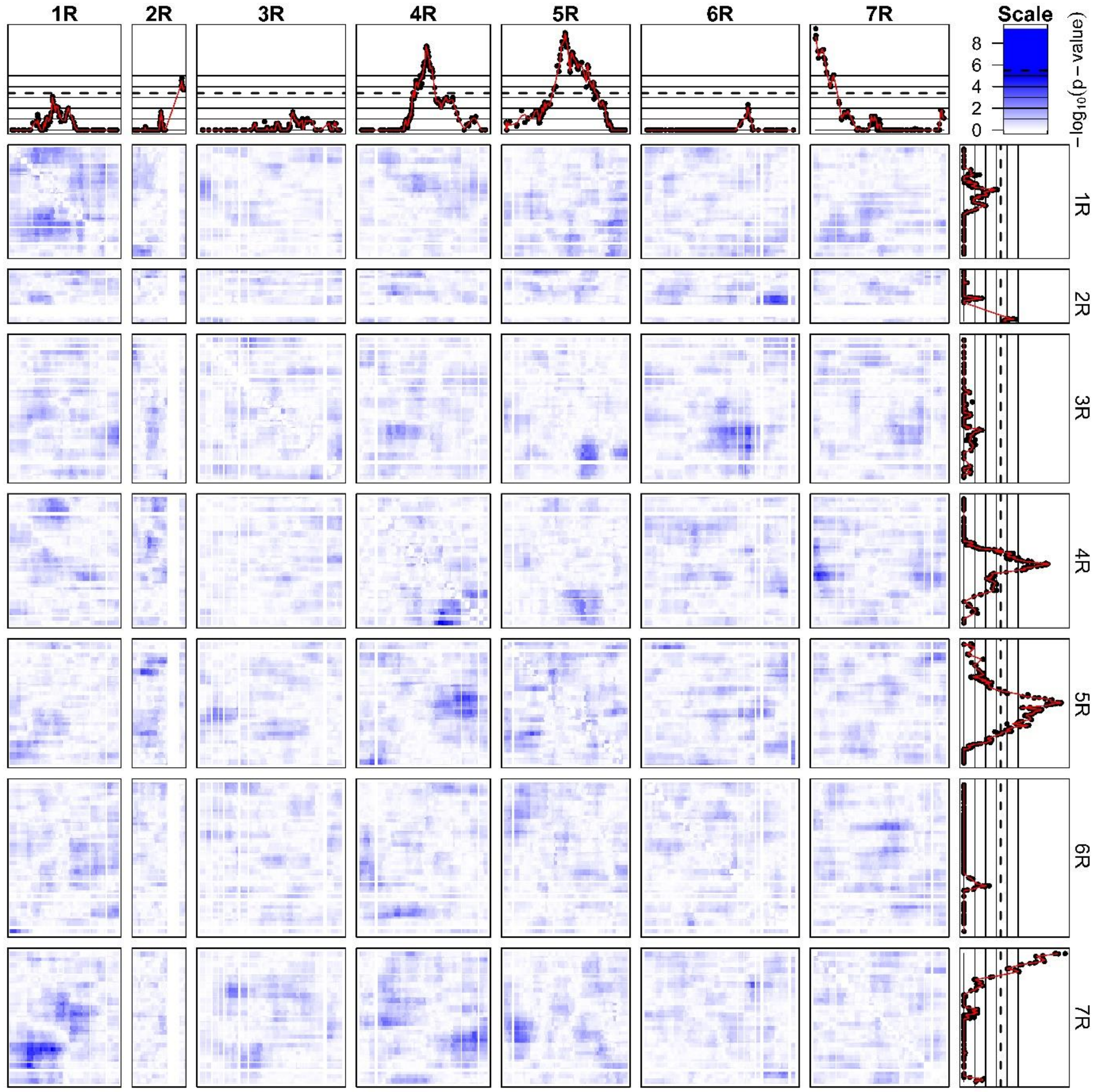

Figure S11 Display of codominant-dominant marker-marker interaction of mapping perenniality. Each box with shades of blue corresponds to a chromosome-chromosome combination and the color scale with the respective LOD score (-log$_{10}$(p-value)) is shown in the upper right corner. This is legend box shows also the axis scale for the LOD-curves of the single marker fits (codominant + dominant combined without cofactors). Note, that this display is non-symmetric. Comparisons in the lower-left triangle of the full plot are based on codominant marker1 in the columns with dominant marker 2 in the rows, and the upper-left triangle is arranged vice versa. Continuous horizontal lines are plotted for better visualization in LOD curves and color legend. Dashed lines are plotted at the level of the global significance threshold. The estimation of the marker-marker interactions was based on a model with fixed marker effects in the following sequence: m1_codominant + m1_dominant + m2_codominant + m2_dominant + m1_codominant: m2_codominant + m1_dominant: m2_dominant + m1_dominant: m2_codominant, where codominant markers were coded 0,1,2 (A,H,B) and dominant markers were coded 0,1,0 (A,H,B).

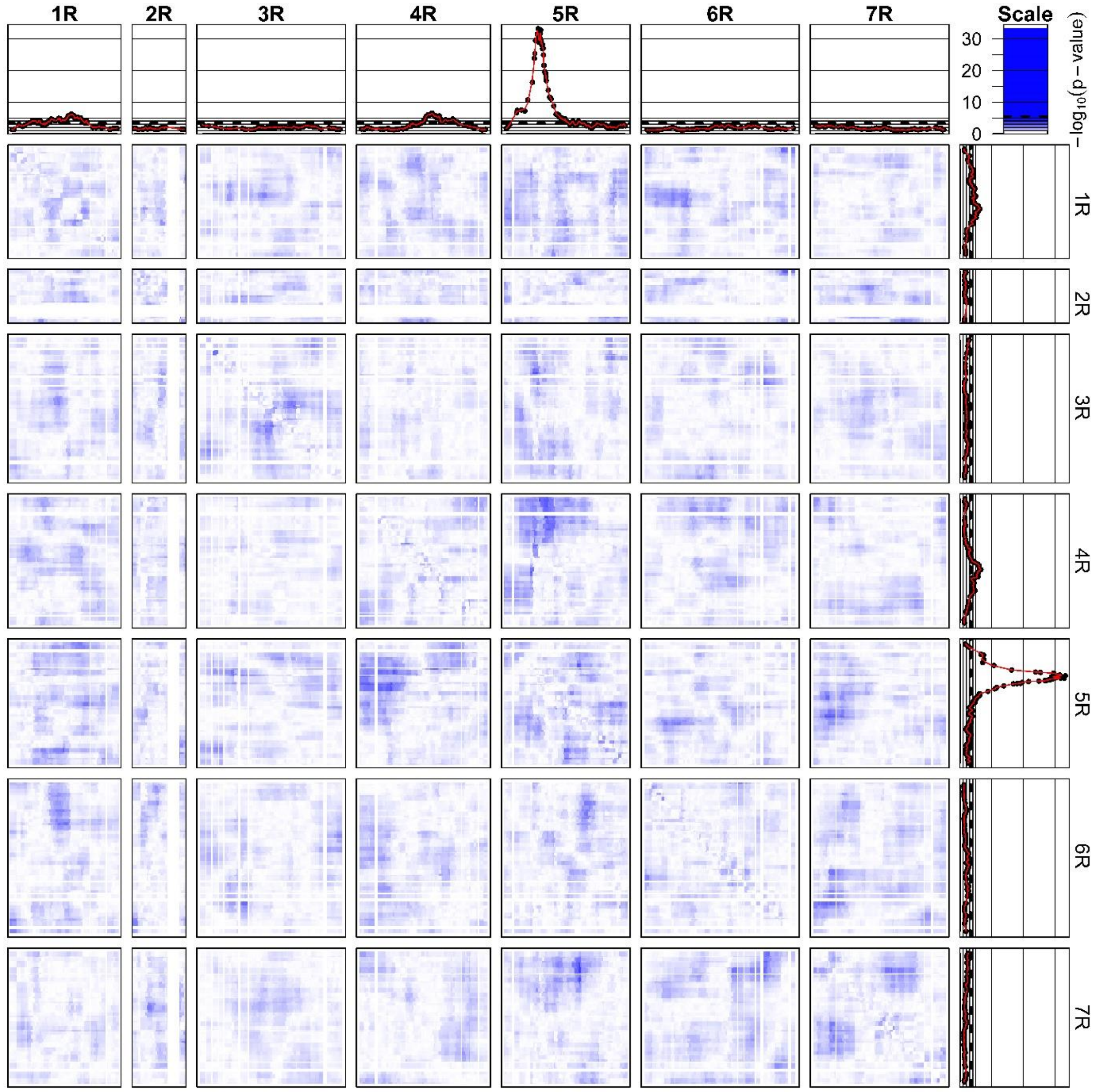

Figure S12 Display of codominant-codominant marker-marker interaction of mapping fertility. Each box with shades of blue corresponds to a chromosome-chromosome combination and the color scale with the respective LOD score (-log10(p-value)) is shown in the upper left corner. This is legend box shows also the axis scale for the LOD-curves of the single marker fits (codominant + dominant combined without cofactors). The display is symmetric, so that comparisons in both directions result can be made. Continuous horizontal lines are plotted for better visualization in LOD curves and color legend. Dashed lines are plotted at the level of the global significance threshold. The estimation of the marker-marker interactions was based on a model with fixed marker effects in the following sequence: m1_codominant + m1_dominant + m2_codominant + m2_dominant + m1_codominant: m2_codominant + m1_dominant: m2_dominant + + m1_dominant: m2_codominant + m1_codominant: m2_dominant, where codominant markers were coded 0,1,2 (A,H,B) and dominant markers were coded 0,1,0 (A,H,B).

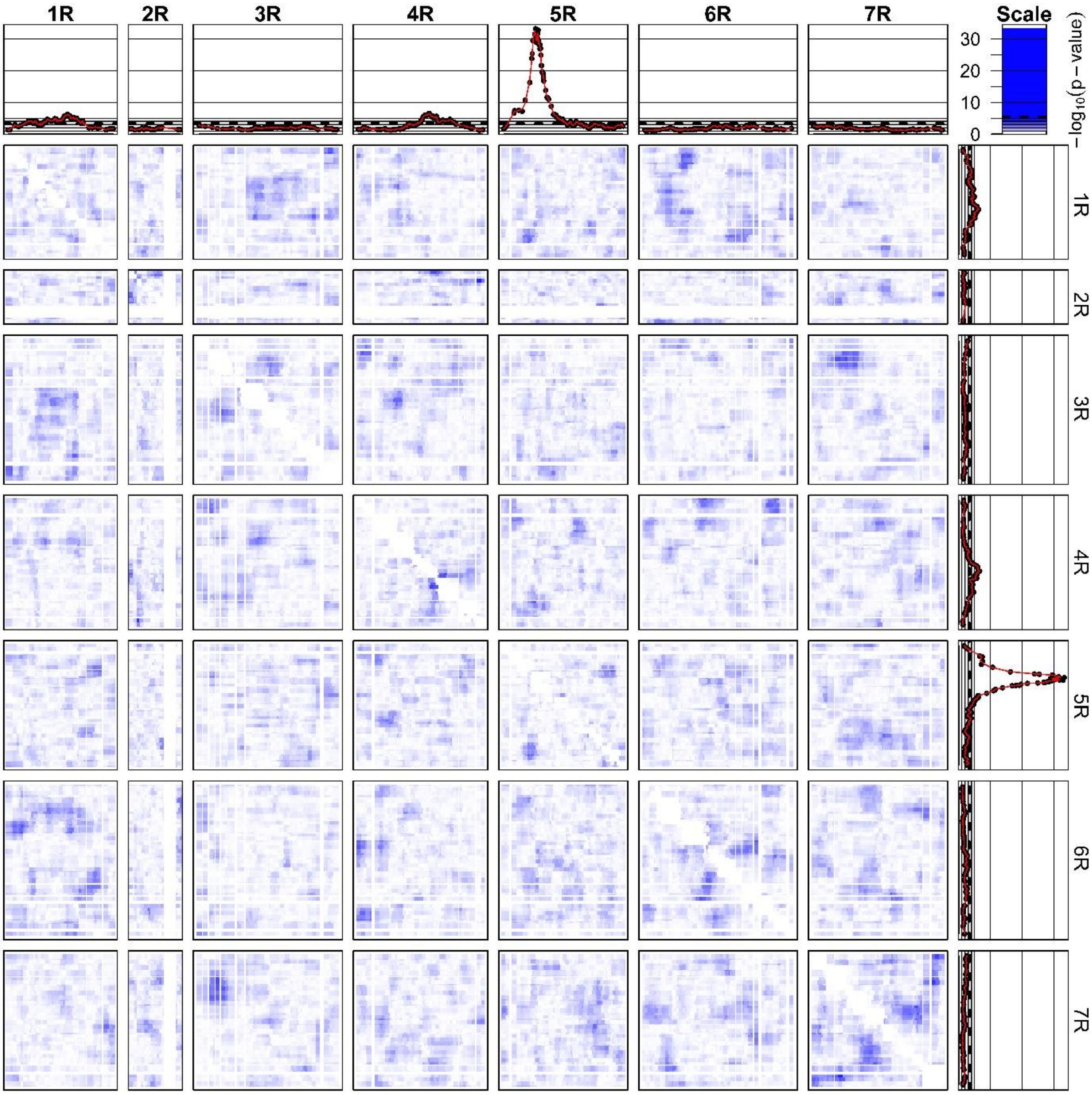

Figure S13 Display of dominant-dominant marker-marker interaction of mapping fertility. Each box with shades of blue corresponds to a chromosome-chromosome combination and the color scale with the respective LOD score (-log10(p-value)) is shown in the upper right corner. This is legend box shows also the axis scale for the LOD-curves of the single marker fits (codominant + dominant combined without cofactors). The display is symmetric, so that comparisons in both directions result can be made. Continuous horizontal lines are plotted for better visualization in LOD curves and color legend. Dashed lines are plotted at the level of the global significance threshold. The estimation of the marker-marker interactions was based on a model with fixed marker effects in the following sequence: m1_codominant + m1_dominant + m2_codominant + m2_dominant + m1_codominant: m2_codominant + m1_dominant: m2_dominant + m1_dominant: m2_codominant, where codominant markers were coded 0,1,2 (A,H,B) and dominant markers were coded 0,1,0 (A,H,B).

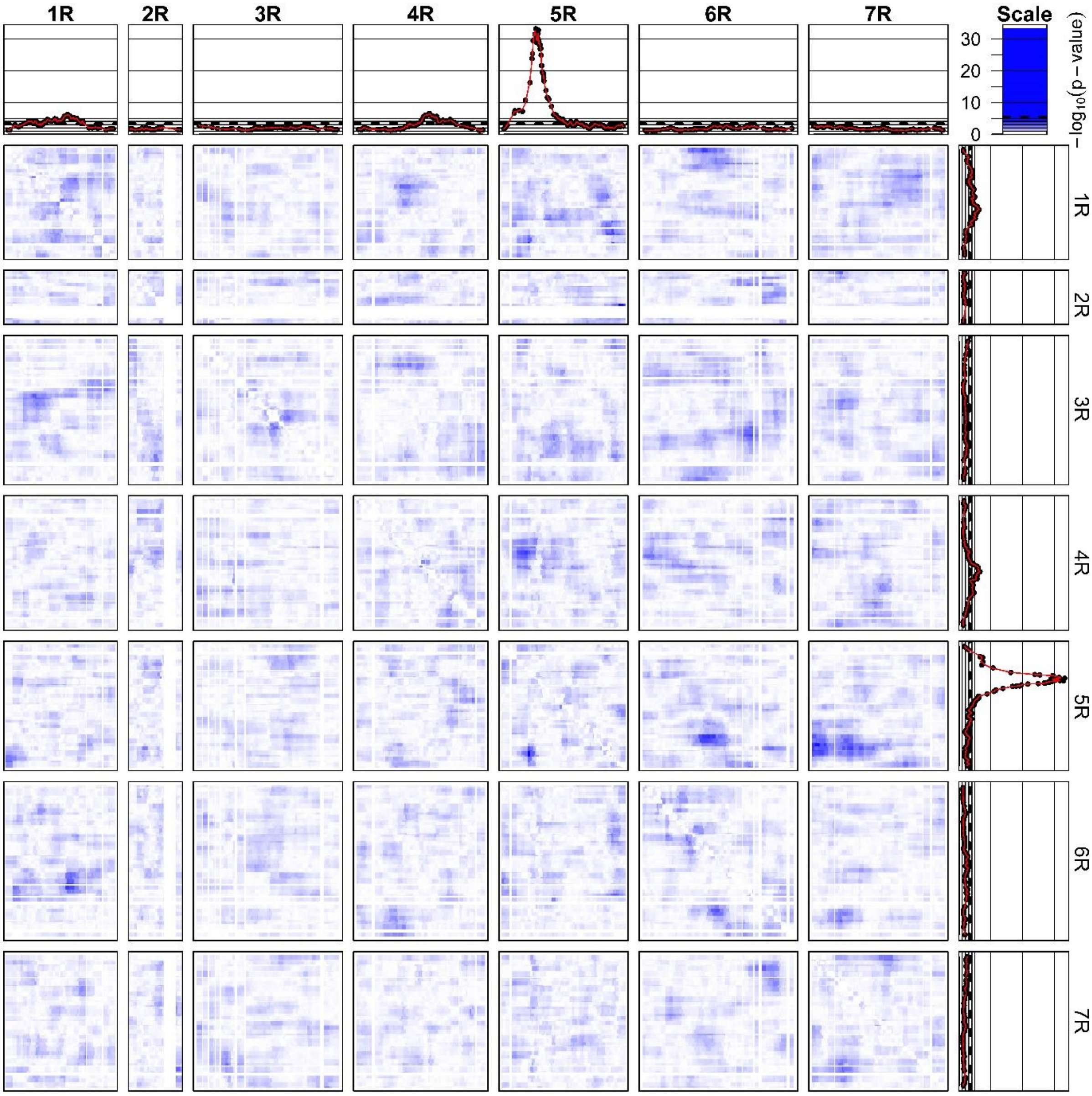

Figure S14 Display of codominant-dominant marker-marker interaction of mapping fertility. Each box with shades of blue corresponds to a chromosome-chromosome combination and the color scale with the respective LOD score (-log10(p-value)) is shown in the upper right corner. This is legend box shows also the axis scale for the LOD-curves of the single marker fits (codominant + dominant combined without cofactors). Note, that this display is non-symmetric. Comparisons in the lower-left triangle of the full plot are based on codominant marker1 in the columns with dominant marker 2 in the rows, and the upper-left triangle is arranged vice versa. Continuous horizontal lines are plotted for better visualization in LOD curves and color legend. Dashed lines are plotted at the level of the global significance threshold. The estimation of the marker-marker interactions was based on a model with fixed marker effects in the following sequence: m1_codominant + m1_dominant + m2_codominant + m2_dominant + m1_codominant: m2_codominant + m1_dominant: m2_dominant + m1_dominant: m2_codominant, where codominant markers were coded 0,1,2 (A,H,B) and dominant markers were coded 0,1,0 (A,H,B).
